# Neural correlates of uncertainty processing in psychosis spectrum disorder

**DOI:** 10.1093/braincomms/fcaf073

**Published:** 2025-02-17

**Authors:** Sophie Pauline Fromm, Lara Wieland, Alix Deneault, Andreas Heinz, Teresa Katthagen, Florian Schlagenhauf

**Affiliations:** Department of Psychiatry and Neuroscience, Charité-Universitätsmedizin Berlin, Corporate Member of Freie Universität Berlin, Humboldt-Universität zu Berlin, and Berlin Institute of Health, CCM, Berlin 10117, Germany; Department of Psychology, Humboldt-Universität zu Berlin, Berlin 12489, Germany; Einstein Center for Neurosciences Berlin, Charité—Universitätsmedizin Berlin, Berlin 10117, Germany; Department of Psychiatry and Neuroscience, Charité-Universitätsmedizin Berlin, Corporate Member of Freie Universität Berlin, Humboldt-Universität zu Berlin, and Berlin Institute of Health, CCM, Berlin 10117, Germany; Einstein Center for Neurosciences Berlin, Charité—Universitätsmedizin Berlin, Berlin 10117, Germany; Bernstein Center for Computational Neuroscience, Berlin 10115, Germany; Department of Psychiatry and Neuroscience, Charité-Universitätsmedizin Berlin, Corporate Member of Freie Universität Berlin, Humboldt-Universität zu Berlin, and Berlin Institute of Health, CCM, Berlin 10117, Germany; Department of Psychiatry and Neuroscience, Charité-Universitätsmedizin Berlin, Corporate Member of Freie Universität Berlin, Humboldt-Universität zu Berlin, and Berlin Institute of Health, CCM, Berlin 10117, Germany; Department of Psychiatry and Neuroscience, Charité-Universitätsmedizin Berlin, Corporate Member of Freie Universität Berlin, Humboldt-Universität zu Berlin, and Berlin Institute of Health, CCM, Berlin 10117, Germany; Department of Psychiatry and Neuroscience, Charité-Universitätsmedizin Berlin, Corporate Member of Freie Universität Berlin, Humboldt-Universität zu Berlin, and Berlin Institute of Health, CCM, Berlin 10117, Germany; Einstein Center for Neurosciences Berlin, Charité—Universitätsmedizin Berlin, Berlin 10117, Germany; Bernstein Center for Computational Neuroscience, Berlin 10115, Germany; NeuroCure Clinical Research Center, Charité—Universitätsmedizin Berlin, Berlin 10117, Germany

**Keywords:** belief updating, psychosis, fMRI, computational modelling, reinforcement learning

## Abstract

Psychotic beliefs are typically held with high certainty. Altered computation of uncertainty about a belief and about environmental dynamics may be an underlying mechanism of psychotic symptoms. We set out to shed light on behavioural and neural correlates of uncertainty processing and how it drives belief updating in psychosis. This cross-sectional study included 19 participants with psychosis spectrum disorder (5 female and 14 male) and 40 healthy control participants (21 female and 19 male) between 18 and 65 years of age. Participants performed a predictive inference task that required belief updating of a noisy outcome in a suddenly changing environment during functional magnetic resonance imaging. Behavioural and imaging data were analysed with a computational model that approximates an ideal Bayesian observer. The model expects beliefs to be updated based on the relative belief uncertainty and environmental change point probability. Task performance, model parameters and associated neural activation were compared between groups and associated with self-reported delusional ideation and cognitive functioning. While the belief updating speed overall did not differ between groups, the psychosis group showed lower task performance. Lower performance was associated with higher self-reported delusional ideation, even when controlling for cognitive functioning. Persons with psychosis spectrum disorder tended to persevere on beliefs after large prediction errors that signal environmental changes. They informed belief updates less by the probability of environmental change points, although this capacity seemed to depend on general cognitive functioning. The psychosis group also encoded the change point probability less in the superior occipital and fusiform gyrus, as well as a cluster comprising pre-central to middle frontal gyrus. Activity in these clusters was associated with lower self-reported delusional ideation across the whole sample and lower general and negative symptoms in the clinical sample. Persons with psychosis spectrum disorder did not seem to overestimate environmental volatility in general. Instead, they showed altered processing of information that occurred after environmental change points, whose probability was less well represented in brain regions encoding visual surprise and motor responses. Possibly, persons with psychosis spectrum disorder inadequately integrated visual surprise signals, leading to ineffective transmission to motor regions that eventually guide behaviour. Summarizing, our study suggests that delusions could result from a tendency to stick to old beliefs even in the light of contrary evidence, due to a failure to integrate uncertainty information based on inferred environmental dynamics.

## Teaser

Using behavioural analysis and computational model-based MRI, Fromm *et al*. investigated behavioural and neural correlates of uncertainty processing and belief updating in psychosis. They conclude that delusions could result from a failure to track environmental change points and uncertainty, accompanied by altered encoding of this information in the brain.

## Graphical Abstract

**Graphical Abstract:**
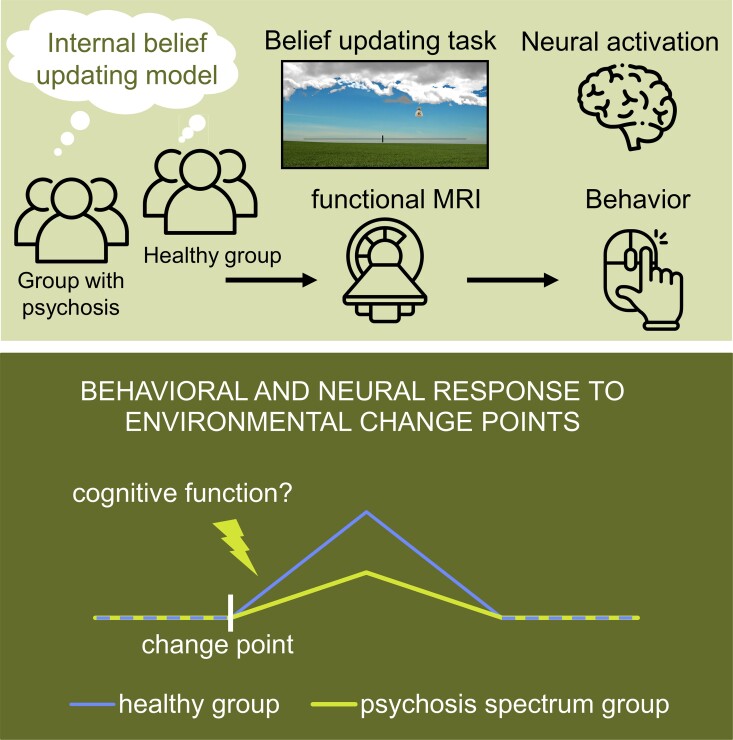

## Introduction

Psychosis refers to the presence of delusions or hallucinations with impaired insight.^1^ Delusions are beliefs and hallucinations are percepts that people with psychosis abide to with high certainty despite contrary evidence. To qualify as delusion, a belief must fulfil the criteria (i) certainty, (ii) incorrigibility and (iii) impossibility or falsity of content.^2^ Thus, high certainty and uncompromising conviction of these mental representations seem to be at the root of psychotic symptoms.^3^ The uncertainty about a belief impacts to what degree it is updated, so that certain beliefs are not updated as quickly as uncertain beliefs. Crucially, belief updating according to subjective uncertainty seems to be associated to dopaminergic neurotransmission.^4,5^ Dopamine functioning is altered in psychosis^6^ and D2 receptors are a main target of antipsychotic medication, which is considered first line treatment to ameliorate psychotic symptoms.^7^ As such, it is biologically plausible that impaired dopaminergic functioning may underlie psychotic symptoms, possibly via altered belief updating and encoding of uncertainty.

**Figure 1:**
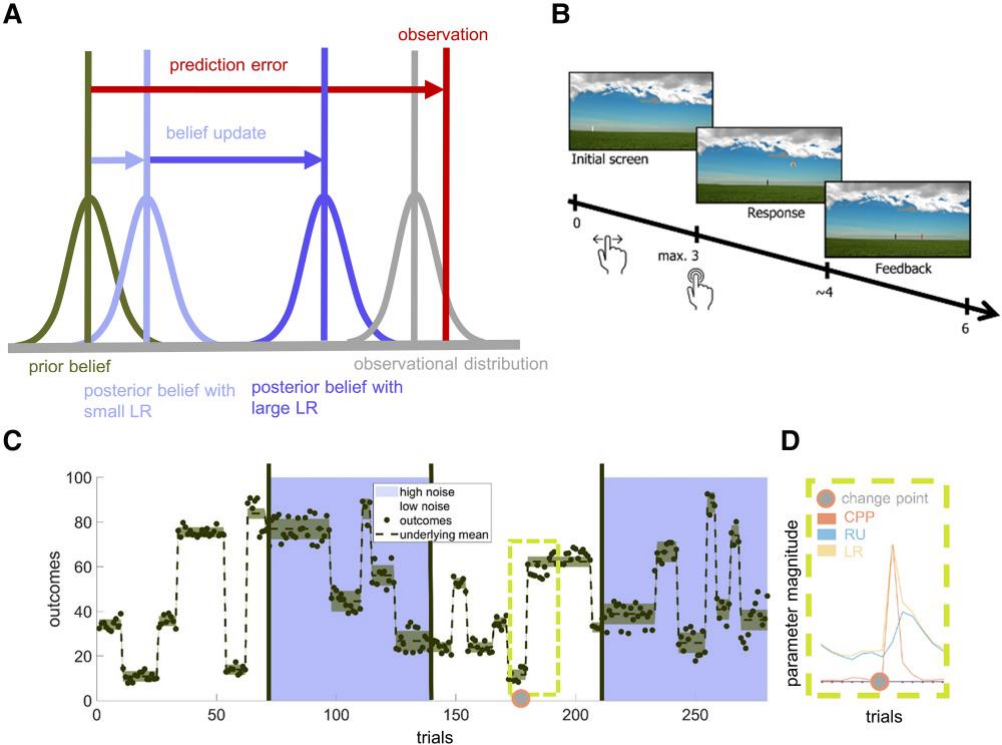
**Illustration of Bayesian belief updating and task and model characteristics. (A)** Illustration of Bayesian belief updating and low versus high learning rate (LR). **(B)** Example trial. **(C)** Trial structure. **(D)** Trajectory of change point probability (CPP), relative unvertainty (RU) and LR at an environmental change point. The cut-out is shown in Fig. 1C, with trials on the *x*-axis and parameter magnitude on the *y*-axis.

So far, the exact behavioural and neural computations underlying belief updating in psychosis are not fully understood. To tap into these computational mechanisms, learning models have been adopted and refined in Bayesian accounts of psychosis.^8–11^ These models rely on the assumption that human beliefs about the world are shaped by prior experience and updated based on new observations. They assume that the magnitude of the belief update is determined by the deviance of the prior belief and the new observation (Fig. 1A). This deviance, also called prediction error (PE), is scaled by the learning rate (LR) that determines to what extent an agent integrates a new piece of evidence to form the posterior belief. As such, the same PE coupled with a small LR results in a small update of the prior belief (Fig. 1A, light purple curve), whereas a larger LR can provoke almost an entire neglect of the prior belief in favour of the new observation (dark purple curve).

The present study adopted a computational model that approximates optimal Bayesian learning.^12^ This model assumes that the LR is dictated by two types of uncertainty, the relative uncertainty (RU) of the outcome belief and the probability that the environment changed. When the environment is stable, the LR is dictated by RU that declines slowly with the number of observations after an environmental change (Fig. 1C and D). In our task, this refers to the RU about the location of the helicopter. Upon a sudden environmental change, which would equal a complete change of the helicopter (position, i.e. change of the underlying mean), the LR is dictated by the change point probability (CPP), allowing flexible updating via rapid upscaling of the LR.^12^ Thus, CPP refers to the probability that the helicopter changed its location completely. The plausibility of the RU- and CPP-trajectories can be illustrated as follows: imagine starting a new job and figuring out the best way to work. Initially, you are highly uncertain if Privet Road, Lilac Walk or Laurel Street takes you to your work place fastest. You try all routes and learn that Privet Road is the quickest. Your initially high RU regarding the belief about the best route slowly decreases due to a gain of experience. After a week, you established a stable belief about the best way to work, and after a year you would never take Lilac Walk or Laurel Street, even if there is a traffic jam on Privet Road once in a while. One morning however, you recognize a sign announcing intermittent construction work on Privet Road. Henceforth, on every morning, you infer from traffic density if construction has started and blocks the route. Soon you recognize traffic jam on Privet Road. This environmental change renders your prior beliefs invalid and requires increasing the LR quickly to allow disregarding the old belief. You must rapidly update your belief about the best route. Now again, you have a high RU about which of the two other options is preferred and you start exploring again. This illustrates that optimal updating behaviour in suddenly changing environments, requires both, learning slowly by adapting towards RU of your belief and scaling up the LR rapidly upon environmental change points.

While this model provides an established framework to examine belief updating, the exact alterations underlying psychotic symptoms remain to be elucidated. The aberrant salience account poses^13,14^ that aberrant dopaminergic firing gives rise to false-positive salience signals, leading to a general tendency to overweigh new evidence or a particular tendency to interpret small prediction errors (PEs) as significant environmental change points. Accordingly, prior reward learning studies across the clinical and non-clinical psychosis spectrum report lower belief stability, a tendency to more rapidly switch between choice options and faster learning from small PEs.^15–20^ Also, people with psychosis or high paranoia seem to overestimate the environmental volatility.^15,16,18,21,22^ Yet, these learning alterations are in contrast to the definition of delusions as highly rigid and resistant towards new evidence. Rigidity would be expressed by slower learning and perseverance on prior beliefs, despite environmental changes. Concordantly, also higher perseverance on beliefs in reinforcement^23–25^ and cognitive^26–28^ learning tasks was shown in people with schizophrenia, hypothetically because dopaminergic PEs vanish in the noise of phasic dopaminergic transmission.^17^ Evidence from perceptual belief updating tasks draws a more nuanced picture, suggesting that it depends on the cognitive level of a belief (ranging from sensory ‘low-level’ towards more abstract ‘high-level’ beliefs), which is examined, whether beliefs are more stable or more flexible in people with delusions.^20^ Taken together, behavioural results indicate that belief updating alterations in psychosis spectrum disorder (PSD) are complex, depend on the relative belief uncertainty of the outcome (RU) and uncertainty related to environmental dynamics (CPP).

The neural encoding of belief updating processes is a topic of recent research. It has been established that PEs are encoded in the cingulate, ventral and dorsomedial PFC, the striatum, midbrain and insula.^29^ Aberrant neural PE-signals were relatively consistently reported in PSD in fronto-striatal regions and aberrations were in some studies correlated with the severity of delusions.^30–35^ More recently, it has been suggested that not only the encoding of the PE-magnitude is altered in psychosis, but also the LR that scales the PE-signal and determines the degree of integration to form the new belief. As mentioned above, the LR is largely driven by the uncertainty associated with the outcome belief and the environment. Neuroimaging studies suggest impaired encoding of belief uncertainty and environmental volatility in the insula, midbrain as well as pre-frontal, temporal and visual cortical regions across the psychosis spectrum.^15,21,36,37^

So far, neural correlates of RU and CPP in the present belief updating paradigm were only investigated in healthy subjects.^38^ The present study leverages on prior behavioural findings^25,36^ and applies the same approximately Bayesian model for optimal belief updating and predictive inference task paradigm during functional magnetic resonance imaging (fMRI). We investigated whether persons with PSD systematically deviate from optimality by altered scaling of LR according to RU and CPP. Our research questions extend previous behavioural findings by exploring how RU and CPP are represented in the brain and how this relates to cognitive functioning and psychotic symptomatology. A stronger relationship with psychotic symptomatology beyond overall cognitive functioning would strengthen the assumption that belief updating is a mechanism underlying psychotic symptoms and not only related to broader cognitive deficits. We expected that CPP and RU are less well encoded on the neural level in PSD, since previous studies showed that these parameters informed behavioural belief updating to a lower extent.^25,36^ Since psychosis has often been related to altered reward processing^39^ and it was shown that learning is faster for rewarded outcomes,^40^ we also examined how PSD versus control participants used reward information to update beliefs. In our task, the reward information is irrelevant for optimal task performance, since a rewarding outcome does not give more reliable information about the next outcome as compared to a non-rewarded outcome. Thereby, we could assess, whether persons with PSD show increased responding to rewarding, but task-irrelevant stimuli, which would eventually impede optimal belief updating.

## Materials and methods

### Participants

We screened data from 60 healthy participants and 38 participants with PSD. A flow-chart showing the number of eligible participants and reasons for exclusion is presented in the Supplementary Fig. S1. The reported sample included 40 healthy control participants (HC, 21 female and 19 male) and 19 participants (PSD, 5 female and 14 male) with diagnosed PSD (all F.2x diagnoses or substance-induced psychosis F10.5-F19.5). Gender of the groups did not differ significantly (χ^2^ = 2.6, *P* = 0.07), but HC were younger than PSD (Table 1). Eligibility was assessed by trained researchers via interview. All participants were required to be between 18 and 65 years old, MRI compatible and fluent in the German language. Participants were recruited between January 2020 and July 2023. HC were recruited via flyers in public spaces in Berlin, Germany and via online advertising, internal email lists and social media groups. Exclusion criteria for HC were any psychiatric disorder according to The Structured Clinical Interview for DSM-5—Clinical Version, neurological diseases, current severe or chronic medical condition, drug abuse or consumption within the past 14 days and first-degree relatives with PSD. Participants in the clinical sample were recruited from in- and out-patient units of the Charité Universitaetsmedizin Berlin Psychiatry and Psychotherapy department. Persons with PSD underwent an interview-based psychopathological assessment of symptomatology, social impairment and current medication. The average dose of antipsychotic medication was 6.46 ± 8.14 mg Olanzapin equivalents. Within the past 6 months seven participants with PSD were only in outpatient care, 10 subjects were treated in inpatient care once, one subject twice and one subject three times. The average duration of illness was 9.73 ± 9.66 years. In order to participate, persons with PSD were required to be clinically stable and to not fulfil criteria for current substance-use disorder. The PSD group included 13 participants diagnosed with schizophrenia (ICD-10, F20), one person with schizoaffective disorder (F25), one person with psychotic disorder due to use of stimulants (F15.5), three persons with psychotic disorder due to multiple drug use and use of other psychoactive substances (F19.5) and one person with acute and transient psychotic disorder (F23.9). We obtained written informed consent from all participants. Ethical consent was obtained by the Charité Ethics committee and adheres to the declaration of Helsinki (20.02.2020/EA1/015/20).

**Table 1:** Participant characteristics and task behaviour

	HC (*N* = 40)		PSD (*N* = 19)		Group difference
	Mean	SD	Mean	SD	*t*-tests
Age (years)	29.3	11.38	35.8	9.67	***t*** **=** **2.15, *P*** **<** **0.04**
Symptomatology					
Self-reported delusional ideation (PDI)	2.9^a^	3.53	14.68	10.04	***t*** **=** **5.48, *P*** **<** 0**.001**
PANSS positive symptoms			16.32	6.98	
PANSS negative symptoms			11.32	3.21	
PANSS general psychopathology			28.63	6.44	
Cognitive performance					
Verbal memory	54.5	7.6	45.2	11.8	***t*** **=** **−3.18, *P*** **=** **0.004**
Working memory	21.3	3.6	29.6	4.3	*t* = −0.77, *P* = 0.450
Motor speed	78.7	13.5	76.6	14.7	*t* = −0.55, *P* = 0.585
Verbal fluency	28.2^a^	6.9	27.8	8.0	*t* = −0.18, *P* = 0.857
Attention	36.5^a^	17.5	24.4	7.26	***t*** **=** **−3.69, *P*** **<** **0.001**
Information processing speed	65.1^a^	13.7	52.9	10.3	***t*** **=** **−3.75, *P*** ***<*** **0.001**
Executive functioning	18.1	2.02	17.6	2.52	*t* = −0.79, *P* = 0.438
Task behaviour					
Missing trials	4.23	7.08	4	4.46	*t* = 0.15, *P* = 0.883
Performance	6.38	0.65	7.33	1.77	***t*** **=** **−2.27, *P*** **=** **0.034**
LR	0.63	0.39	0.57	0.41	*t* = −1.38, *P* = 0.176
LR after change point	0.84	0.08	0.75	0.13	***t*** **=** **2.96, *P*** **=** **0.007**
Non-updates	21.1%		28.5%		*t* = 1.99, *P* = 0.056
Moderate updates	39.6%		35.1%		*t* = −1.51, *P* = 0.139
Total updates	39.4%		36.4%		*t* = −0.69, *P* = −0.494

	Median	SD	Median	SD	Logistic regression
β PE	0.76	0.14	0.73	0.20	β = −5.4, z = −1.8, *P* = 0.072
β CPP	0.15	0.16	0.12	0.19	**β** **=** **−7.05, z** **=** **−2.33, *P*** **=** 0**.02**
β RU	0.11	0.39	0.26	0.64	β = 1.87, z = 1.48, *P* = 0.138
β reward	0.03	0.06	0.02	0.14	β = −2.52, z = −0.6, *P* = 0.551

^a^Data of one healthy subject is missing.

### Clinical and cognitive assessment

Upon the first appointment, all participants provided basic demographic and clinical data. All participants completed Peters Delusion Inventory.^41^ The scale was developed to assess delusional ideation in non-clinical populations. Reported analysis was conducted upon the number of endorsed items. In PSD, trained researchers administered the positive and negative syndrome (PANSS) scale for schizophrenia.^42^ We computed symptom severity on the positive, negative and general psychopathology scale of the PANSS. Cognitive functioning was assessed with the brief assessment for cognition in Schizophrenia^43^ (BACS, Keefe *et al*., 2004) by trained researchers upon the first appointment. The cognitive battery comprises six sub-domains including (i) verbal memory (list learning), (ii) working memory (digit sequencing task), (iii) motor speed (token motor task), (iv) verbal fluency (category instances and controlled oral word association test), (v) attention and speed of information processing (symbol coding) and (vi) Executive functions (Tower of London).

### Predictive inference task

To investigate belief updating, we administered a predictive inference task during fMRI scanning. Variants of this task were previously used in various clinical and non-clinical samples^25,44,45^ and administered during fMRI scanning.^38^ The task was displayed on a back projection screen that participants could view via mirrors attached to the head coil. Participants navigated the task and indicated their decisions using a trackball device. The task requires participants to track a dynamic hidden mean and differentiate sudden change points from noisy observations. The target is represented by a helicopter and environmental change points are represented by a change of location of the helicopter. Participants were instructed that they could maximize rewards by catching bags dropped from the helicopter (instructions in Supplementary material S2). Participants were informed that the bags were slightly blown aside by wind, so that the exact location of the bags noisily fluctuates. Critically, it was known to participants that the helicopter usually stayed in one place and upon unannounced environmental change points, relocated completely. As shown in Fig. 1D, CPP increases upon such an environmental change point and quickly drops right after, whereas RU is at peak on the trial after the environmental change point and decreases slowly with the number of observations until the next change point occurs.

An example trial is depicted in Fig. 1B. Participants first saw a fixation cross, followed by a screen with a landscape indicating the beginning of the decision period. Upon indication of their belief of the helicopter location on a scale (0–100), the bag dropped. The bag was labelled to contain either sand or money, which was unknown to participants until after their response. Participants were informed that received a financial bonus if they caught money bags. A bag was considered caught when the mouse was at the location where the bag dropped ± 5, which equals the width of the bag image on the screen. During the feedback period, they received feedback about whether they caught the bag indicated by a sound and the bag sticking longer on the screen. The PE was indicated on the screen with a red line, showing how far the prediction deviated from the actual location where the bag dropped. After a jittered inter-trial-interval with an average duration of two seconds, a new trial started with the fixation period.

After practice inside and outside the scanner, participants performed 280 experimental trials split into four runs with 70 trials each. In the first and third run, the position of the bag fluctuated weakly around the underlying helicopter location (noise = 2.3 screen units). In the second and fourth run, the position fluctuated stronger (noise = 4.6 screen units), which renders accurate tracking of the helicopter more difficult. The trajectory of the helicopter, including rewarded outcomes, as well as the change points and all locations, where bags dropped was fixed across participants (Fig. 1C). Data of one subject was excluded due to missing values on more than 25% of the trials and of one subject who showed non-compliance with task instructions.

### Statistical analysis of the behaviour and computational model

Belief updates were quantified as the difference between the participants’ prediction from one trial to the next. PEs’ were computed as the deviance between the prediction and the observed outcome. Performance was quantified as the summed deviance between the prediction and the underlying mean (performance error) of the current block. We compared the groups regarding their mean performance aggregated across all trials using a *t*-test and correlated performance with general cognitive functioning scores. Performance was entered into a linear regression predicting PDI, while controlling for general cognitive functioning.

One block is defined as trials with a common underlying mean with blocks being separated by change points. We computed dynamic LRs that fluctuate across trials by dividing the belief update of the current trial by the PE of the previous trial. Compared to fixed LRs, this allows investigating how belief updating is adjusted across time and according to environmental dynamics. LRs larger than one were set to one and negative LRs were set to zero. LRs larger than one represent belief updates far beyond and negative LRs represent updates in the opposite direction of the previous observation. This may be a form of non-optimal learning, but most likely occurs due to a lapse of attention or an aberrant hand movement. Although data aggregation depresses this information, we tried to mitigate the impact of these trials by these categorizations similar to Nassar and colleagues.^25,36^ We compared average LRs aggregated across trials between the HC and PSD groups, as well as LRs on trials after environmental change points. To examine extreme belief updating, we categorized LRs as non-updates (LR ≤ 0.01), moderate updates (0.1 < LR > 0.09) and total updates (LR ≥ 0.9) and compared the update fractions (proportion of each category in all trials) between the groups similar to previous studies.^25,36^ We visually inspected individual choice data to ensure participants adhered to the task and did not show aberrant behaviour. Examples of aberrant behaviour are random predictions that seem to not follow the outcome trajectory at all or repetitive behaviour, like the same prediction for all trials. The visual output of an included and an excluded subject that showed random predictions are shown in the Supplementary Figs. S2 and S3.

The behavioural belief updating data of each participant was modelled individually using a computational model, derived from the fully Bayesian ideal observer.^12,25,38^ This model approximates the optimal predictive distribution via a Gaussian distribution with a matched mean and variance. According to this model, optimal behaviour requires mean tracking of the helicopter location instead of exact following of the bag location, and adjusting the LR according to RU about the bag location as well as to CPP. As such, the model computes trajectories of CPP and RU based on the individual observed PEs. The reward manipulation (money or sandbag) is non-informative for optimal behaviour. CPP peaks if a surprisingly large PE was observed that is unlikely to occur if the environment was stable, therefore indicating an environmental change point. The second factor is RU, which increased on the trial after a change point and slowly decreases as more observations are made within a stable environment. Relevant formulas are reported in Supplementary material S4. Using regression analyses, we explored how well the model parameters predicted our participants’ belief updates. The regression included an intercept (to model a left/rightward tendency), the observed PE magnitudes, individually computed CPP-parameter and RU-parameter, a binary reward coefficient, indicating whether a sand or money bag was observed on the respective trial and a term accounting for the edge effect (bucket location to the power of three), modelling a tendency to avoid the edges of the screen (similar to Nassar *et al*.^25^). All regressors were centred before entering the regression. The CPP-parameter was added as interaction effect with PE and RU was added as interaction with PE and 1-CPP in order to examine the unique effects of each. We assessed whether the regression model fit differed between the groups using a *t*-test.

We applied a logistic regression to predict group status from the model coefficients. Thereby we assessed whether the groups used the information represented by CPP, PE, RU and reward differently to inform the belief updates. We added participant age to evaluate if this improves model fit, since age differed significantly between the groups. Next, we controlled the relationship of performance and model coefficients for general cognitive functioning by adding these to the logistic regression. In addition to the analysis on group status, we fed the model coefficients into a linear regression predicting the total PDI scores across the whole sample and PANSS scores in persons with PSD. This allowed us to examine whether higher (sub-clinical) symptoms were associated with specific parameter alterations in the computational model.

### FMRI acquisition and statistical analysis

Multi-band echo planar images were acquired on a 3T Siemens Trio scanner (Erlangen, Germany) with a 64-channel head coil (repetition time = 800 ms, echo time = 37 ms, voxel size 2 × 2 × 2 mm^3^, flip angle = 52°; 72 axial slices; multi-band factor = 8).^46^ Imaging data were converted into BIDS-format and fed through the fMRI-prep pipeline.^47^ This pipeline automatizes slice-time and head-motion correction, tissue reconstruction via definition of white and gray matter boundaries and co-registration of the functional images to a structural T1-image. Susceptibility distortion correction is applied to account for field inhomogeneity inside the scanner and movement parameters are extracted to be included in first-level statistics. For quality control, each step of the pipeline was visually inspected in the fmriprep output files, controlled for abnormalities and high frame wise displacement, indicating head movements > 5 mm, resulting in exclusion of two subjects. Using SPM12, we conducted further image processing. First, images were smoothed with a Gaussian kernel of 5 mm. Next, first level-statistics were modelled according to a previous study^38^ using an event-related analysis with the general linear model approach as implemented in SPM12 to convolve the onsets with a hemodynamic response function. The first regressor modelled the onset time of the bag drop as an event with one second duration. This onset was parametrically modulated with (i) the outcome location on the screen, (ii) model-based CPP, (iii) model-based RU, (iv) binary reward (money or sand bag) and (v) the trial-wise residual from the computational model. The residual quantifies if participants updated beliefs to a greater or lesser extent than the individual computational model predicts. Thereby, we can account for variability in belief-updating that is not attributable to any of the computational factors, but may instead relate to task-unrelated fluctuations in physical arousal.^38^

Parametric modulators were not orthogonalized, but standardized before being entered to the GLM. A second regressor modelled error trials with a duration of the whole missing trial, when participants did not indicate a response within 3 s after the bag drop. In addition, six nuisance regressors for head movement translation and rotation in *x*, *y* and *z* directions were included. On the second level, we used a flexible factorial design as implemented in SPM12 with the contrast images of the onset and the five parametric modulators for HC and PSD group with age as covariate. Thereby, we could analyse all main effects and group differences of interest within one coherent second-level design. Correction for multiple comparisons was performed using family wise error correction (FWE) *P* ≤ 0.05 at the voxel level and cluster level. In a next step, we extracted effect sizes from clusters that showed significant group differences on CPP and RU and associated the averaged volume of interest (VOI) values with clinical and cognitive outcomes (PDI, PANSS and BACS-scores) using Spearmans rho within R.

## Results

### Behavioural raw data analysis

**Figure 2:**
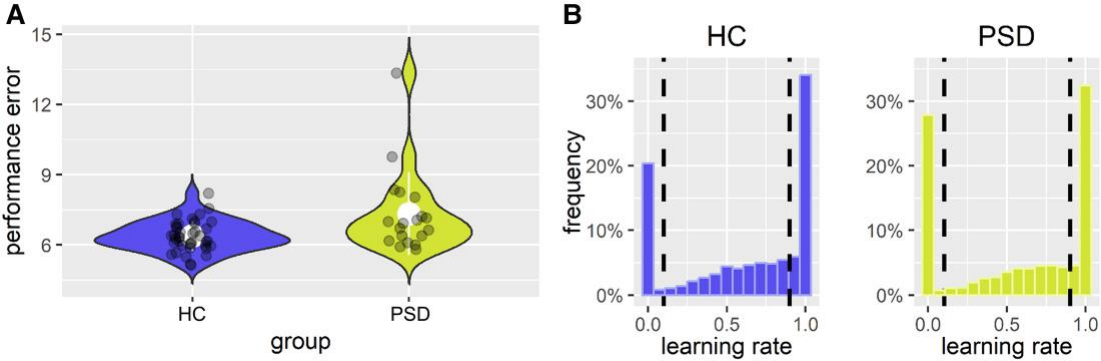
**Behavioural results. (A)** Average performance errors of HC and PSD. Dots represent individual subjects (*n* = 59). **(B)** Fraction of LRs used in the task. Dotted lines mark the categories of non-updates (LR < 0.1), moderate updates (0.1 LR < 0.9) and total updates (LR ≥ 0.9) of beliefs. The left panel shows a histogram of LRs in the HC group (*M* = 0.63, SD *=* 0.39) and the right panel LRs in the PSD group (*M* = 0.57, SD = 0.41). Average LRs did not differ significantly between groups (*t* = −1.38, *P* = 0.176).

HC and PSD missed a similar number of trials (∼4%). Performance errors, which quantify the absolute deviance of the prediction and the underlying mean were larger in PSD (7.33 ± 1.77) as compared to HC (6.38 ± 0.65) (Table 1; Fig. 2A). Wilcoxon rank-test and a repetition of the *t*-test after exclusion of two outliers in the PSD group supported these results (Supplementary material S5). Performance errors in the task correlated with lower cognitive performance (composite z-score of all BACS tasks, *r_ρ_* = −0.43, *P* < 0.001). Examining cognitive sub-domains, performance errors only correlated with domains of verbal memory (*r_ρ_* = −0.46, *P* < 0.001) and information processing speed (*r_ρ_* = −0.45, *P* = 0.001). Performance was a significant predictor for delusional ideation (*β*_Performance_ = 1.98, *P* = 0.046), while controlling for verbal memory and information processing speed (details in Supplementary Table S1).

HC and PSD used overall similar LRs (Table 1). On trials after a change point, when fast updating is optimal, persons with PSD learned more slowly (*t* = 2.96, *P* = 0.007). We categorized all LRs on each trial into groups of non-, moderate- and total-updates. While the fraction of moderate and total updates did not differ between HC and PSD (*P* > 0.1), there was a trend towards more non-updates in PSD (*t* = 1.99, *P* = 0.056), suggesting more perseveration on prior beliefs (Table 1; Fig. 2B). In our follow-up analysis, trials with LRs < −0.1 were excluded since these do not fall in the range of non-updates, but may represent random behaviour. The analysis could not confirm more non-updates in PSD (Supplementary Table S2 and Fig. S4) and indicated that aberrant behaviour, such as large negative LRs, instead of non-updates were increased in PSD. However, by analysing only trials after a change point, we could confirm that persons with PSD learned more slowly (*t* = 2.96, *P* = 0.007) specifically after a change point.

### Computational model analysis

Our computational model analysis indicated that participants made larger updates after they observed large PEs (median β_PE_ = 0.76, SD = 0.16, *t* = 35.6, *P* < 0.001) and learned faster when an environmental change point was likely (median β_CPP_ = 0.14, SD = 0.17, *t* = 7.05, *P* < 0.001) or when they were uncertain about the current belief (median β_RU_ = 0.16, SD = 0.5*, t* = 3.29, *P* = 0.002). Participants also based beliefs on the non-informative factor reward (median β_reward_ = 0.03, SD = 0.09, *t* = 2.45, *P* = 0.017), suggesting that updates were larger after trials that indicated financial bonus.

To assess how PSD and HC differed in the use of the computational updating model, we compared individual regression coefficients and model fit between groups. Distributions of regression parameters per group are displayed in Supplementary Fig. S5. Regression fits (R^2^) were higher in HC (*M* = 0.87, SD = 0.05) as compared to PSD (*M* = 0.78, SD = 0.15, *t* = 2.75, *P* = 0.012), indicating that HC showed less behaviour that cannot be explained by the model parameters, reward or an edge effect. PE-magnitude was a trend-wise predictor (β = −5.4, z = −1.8, *P* = 0.072) and CPP a significant predictor of group status (β = −7.05, z = −2.33, *P* = 0.02), suggesting that updates of persons with PSD were less informed by PE-magnitude and CPP (Table 1). Updating based on RU and reward had no predictive information for group status (*P* > 0.1). We computed pairwise correlations of model coefficients with age. Since age was not significantly correlated with model coefficients (*P* > 0.1), it was not used as covariate in our initial main analysis. In a follow-up analysis when participant age was nevertheless included in the model, PE was no significant predictor of group status anymore (β = −3.82, *P* = 0.24), while CPP (β = −5.55, *P* = 0.089) and RU (β = 1.6, *P* = 0.07) were trend-wise significant predictors, suggesting that PSD used slightly less CPP-information and more RU-information when forming their updates.

Since performance was associated with verbal memory and information processing speed, we controlled for these variables in the analysis of the relationship between model coefficients and group status. The CPP-effect did not remain significant when controlling for verbal memory and information processing speed (Supplementary Table S3).

### Individual differences of behaviour and clinical outcomes

Higher self-reported delusional ideation (PDI) was trend-wise associated with less updating according to CPP (β_CPP_ = −15.76, *P* = 0.084) and significantly more updating according to RU (β_RU_ = 7.14, *P* = 0.003). In PSD, PANSS scores in the positive symptom domain were associated with updating more according to PEs (β_PE_ = 30.83, *P* = 0.006) and more to RU (β_RU_ = 10.41, *P* < 0.001). Scores in the negative symptom domain were not related to model coefficients. PANSS scores on the general psychopathology scale were predicted by higher coefficients for RU (β_RU_ = 7.09, *P* = 0.018) and reward (β_reward_ = 27.97, *P* = 0.048).

### fMRI group statistics

For model-based fMRI analyses, we examined neural activation related to CPP, RU and reward across the whole sample (Supplementary Tables S4–S7 and Figs. S6–S8). CPP was represented in multiple brain areas, such as the post-central and middle frontal gyrus (MFG), anterior insula, thalamus, putamen and cerebellum. Activation related to RU was present in the bilateral cerebellum, supramarginal, pre- and post-central and gyrus, in the superior frontal gyrus and posterior insula. Reward-related activation was present in the bilateral caudate, inferior occipital and fusiform gyrus, posterior orbital and superior frontal gyrus, the anterior insula and anterior cingulate gyrus.

**Figure 3:**
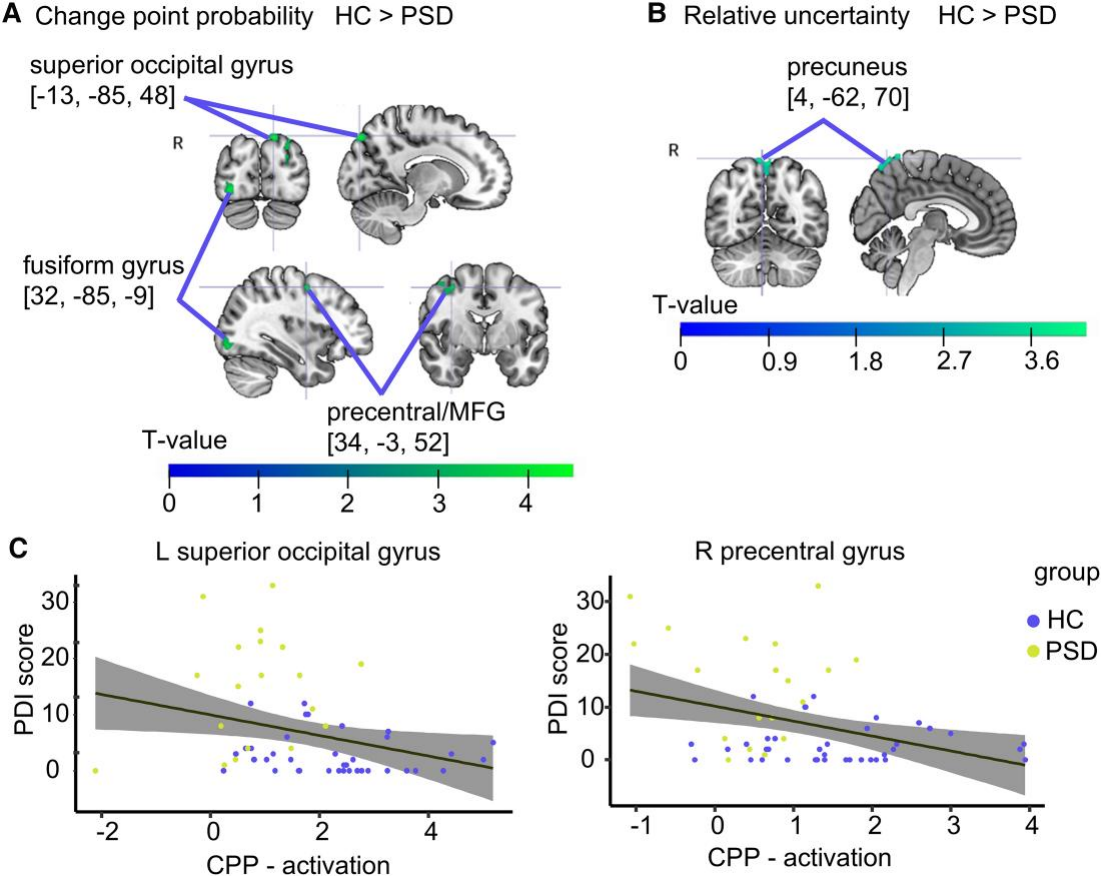
**Neural activation related to CPP and RU.** Clusters are displayed at *P* < 0.001 uncorrected and were significant at the cluster level *P*_FWE_ < 0.05. **(A)** Clusters with significantly activation related to CPP in the contrast HC > PSD. **(B)** Cluster with significant activation related to RU in the contrast HC > PSD. **(C)** Correlation of VOI activation and self-reported delusional ideation (PDI = Peters delusion inventory) in the left superior occipital gyrus (*r***_ρ_** = −0.33, *P* = 0.011) and right pre-central gyrus (*r**_ρ_*** = −0.25, *P* = 0.058). Dots represent individual subjects (*n* = 59). MFG, middle frontal gyrus; R, right; L, left. Colour bar and heat maps represent *t*-statistics.

The neural activation was compared between PSD and HC, while controlling for age. The neural representation of CPP was stronger in HC in three clusters, including the superior occipital (*t* = 4.6, *P_FWE cluster-level_* < 0.001, peak[-13, −85, 48], k = 311) and fusiform gyrus (*t* = 4.24, *P_FWE cluster-leve l_* = 0.028, peak[32, −85, −9], k = 97), and the pre-central gyrus, stretching towards the MFG (*t* = 4.18, *P_FWE cluster-level_* = 0.01, peak[34, −3, 52], k = 119) (Fig. 3A). Neural activation related to RU was stronger in HC in the right precuneus (*t* = 4.37, *P_FWE cluster-level_* < 0.001, [4, −62, 70], k = 249) (Fig. 3B). Activation related to reward did not differ between groups.

### fMRI individual differences

VOI-values were extracted from the three clusters in which CPP-related activation differed between groups and the single cluster that showed group differences related to RU (Fig. 3C). Self-reported delusional ideation was significantly associated with lower CPP-related activation in the left superior occipital gyrus (*r***_ρ_** = −0.33, *P* = 0.011) and trend-wise in the right pre-central gyrus (*r**_ρ_*** = −0.25, *P* = 0.058), but not in the right occipital fusiform gyrus (*r**_ρ_*** = −0.158, *P* = −0.238). Self-reported delusional ideation did not correlate with activation in the RU-cluster (*r**_ρ_*** = −0.04, *P* = 0.744). Lower CPP-related activation in the right occipital fusiform gyrus was related to the PANSS general psychopathology (*r**_ρ_*** = −0.43, *P* = 0.011) and negative symptom domain (*r**_ρ_*** = −0.34, *P* = 0.047), while the other VOI-values were not associated with any of the PANSS scales (*P* < 0.05).

Stronger encoding of CPP in the cluster involving the pre-central to MFG was associated with higher verbal memory (*r**_ρ_*** = 0.31, *P* = 0.017) and processing speed (*r**_ρ_*** = 0.50, *P* < 0.001). Stronger encoding of CPP in left superior occipital gyrus was associated with higher processing speed (*r**_ρ_*** = 0.28, *P* = 0.034). No other cognitive domain was associated with VOI-values and RU was not associated to scores in any cognitive domain.

## Discussion

Delusional beliefs are inflexible towards environmental dynamics and resist contradictory evidence. In line with this, we found that beliefs of patients with PSD were less accurate and more perseverant after meaningful environmental change points. Optimal updating behaviour required tracking the RU of a belief and the probability of environmental change points (CPP) and dynamically adjusting LRs to scale the belief updating signals. Our results suggest that PSD disregard CPP when updating beliefs and represent this information less precisely in the brain. To our knowledge, this study is the first to investigate neural representations of these updating components in people with PSD.

### Learning behaviour

PSD were overall less accurate in tracking beliefs, as indicated by larger performance errors. Also, the degree of self-reported delusional ideations was related to lower accuracy, even corrected for the level of cognitive performance. To examine the underlying dynamics in more detail, we analysed single trial LRs. While the aberrant salience account suggests generally increased LRs, HC and PSD in our study overall used similar LRs. Interestingly, people with PSD tended towards more perseverance on beliefs, especially after environmental change points. This is in line with prior computational modelling studies in patients with schizophrenia, modelling perseverance with a specific parameter that on each trial reduces the LR. These parameters were higher in PSD so that beliefs were stickier and updated slower.^25,26^

Persons with PSD were overall less accurate in tracking beliefs, indicated by higher performance errors. Moreover, the computational modelling analyses showed a trend that the updates of PSD were less attuned to PEs. Much clearer was the finding that PSD did not scale up the learning speed rapidly according to CPP, which rises upon large PEs, indicating that an environmental change took place. This ties in with our previous work relating sub-clinical delusional ideation to less CPP-based belief updating.^36^ As CPP is an estimate of environmental dynamics, the finding also broadly matches studies showing that the estimation of environmental volatility is altered in PSD.^15,16,18,22^ However, we have no direct evidence for a general overestimation of environmental volatility as shown in these studies. Delusional ideation across the whole sample, as well as stronger positive and general psychopathology symptoms in patients, was related to more updating according to RU. This implies that LR-dynamics in people with higher delusional symptoms rather resemble the RU trajectories, which less dynamically react to environmental change points. Taken together, the trend of HC to update more according to CPP, and the relationship between positive symptoms and updating according to RU could reflect that HC more easily identified change points and scaled up learning speed, while people with higher delusional ideation took more time to do so. This results in less responsive belief updating upon environmental change points. What remains to be elucidated is how these indirect behavioural indices of RU and CPP translate to explicit ratings of uncertainty. Seow and Gillan confirmed that low explicit confidence ratings of the outcome belief were related to larger belief updates.^48^ They also report that people scoring high on the trans-diagnostic factor of compulsive behaviour and intrusive thought were overly confident about beliefs but showed lower coupling between actions and confidence. This suggests that they did not use their explicit knowledge about belief uncertainty to inform the updating behaviour, which could equally be the case in the present study.

### Neural activation

The overall pattern of neural activation related to CPP, RU and reward was relatively similar to the activation reported by McGuire and colleagues in healthy subjects.^33^ They showed that CPP was uniquely associated with neural activity in bilateral visual cortices. They speculate that this occurs because CPP may be inferred via the unexpectedness of new sensory representations. In our study, visual cortices in PSD were less active in response to CPP and lower activation was related to higher self-reported delusional ideation across the whole sample. The pre-central gyrus also showed this pattern. The pre-central gyrus is involved in smooth eye pursuit.^49^ This capacity is needed to track moving objects and requires cognitive functions such as attention, selection and prediction.^50^ Deficits in smooth eye pursuit are reported in schizophrenia and close relatives, and were therefore even considered as a candidate endophenotype of schizophrenia.^51^ Thus, group differences in CPP-related activation may reflect a deficit to visually track large shifts of the helicopter location. Taken together, PSD seem to encode the visual-spatial surprise information less clearly, possibly resulting in weaker signal-transmission to other regions implicated in the generation of learning signals and action based on that information.

McGuire showed that activation related to RU was uniquely present in bilateral anterior PFC, parietal cortex and cerebellum.^38^ Among others, we also found activation related to RU in these areas. However, groups differed in activation in the right precuneus, a region known for various functions. Particularly, the dorsal-anterior precuneus is functionally connected to the superior parietal cortex and co-activated during execution or preparation of spatially guided behaviour and mental imagery.^52^ Possibly, control participants expected larger PEs, when RU was high and already prepared to move the trackball device at the time of the feedback. However, it remains to be reconciled how the stronger encoding of RU in HC as compared to PSD could result in less behavioural updating according to this parameter in HC as compared to PSD, when controlling for age. One explanation is that HC are well able to track this information but do not use it in their belief updating model. This would result in stronger encoding, but less updating according to RU.

Two other findings seem at odds at first sight. First, lower occipital CPP-related activation was related to higher general and negative symptoms, but there was no relationship between CPP and symptom scores behaviourally. Second, there was a lack of relationship between self-reported delusional ideation and activation in the RU-cluster, whereas RU-weighting during belief updates was linked to higher PDI. The lack of neural association matching these behavioural findings may be due to a lack of power in our study or due to another mechanism taking place after neural encoding that masks a potential behavioural relationship. Also, we can only speculate why precuneal activation was not related to CPP. Possibly, the (anticipated) movement of the mouse device followed more a RU trajectory that slowly decreases after a change point. Hand movement was needed on every trial. Since CPP scales down to almost zero within a few trials after a change point, this trajectory may not resemble the anticipated hand movement. However, this remains an open question and future studies should examine if other response devices elicit similar activation.

As already mentioned, the neural encoding of RU and CPP were not investigated in PSD until now. A related measure is the so-called precision-weighted PE, a parameter in hierarchical Gaussian filter models that represents an uncertainty-scaled PE.^53^ This precision-weighted PE is similar to the interaction of PE with CPP and RU respectively, which also in our study represents a PE scaled by outcome and environmental uncertainty, although the exact relation between different modelling approaches and model-based fMRI analyses remains to be exactly specified.^10^ Precision-weighted cortical PE signals in the superior frontal cortex were diminished in people with a first psychotic episode.^37^ This aberration was related to positive psychotic symptom severity. On the other hand, Cole *et al*.^21^ found that the encoding of precision-weighted PEs was enhanced in pre-frontal and insular regions of individuals at a clinically high risk for psychosis, similar to results from our work in people with clinical and non-clinical delusions.^36^ It has been reported that dopaminergic regions such as the midbrain and striatum signals guide the update magnitude.^54^ We did not see striatal or midbrain activation related to parameters that determine the degree of updating. Instead, the reward manipulation resulted in activation of the bilateral caudate. This did however not differ between groups, as well as the use of reward information that was non-informative for accurate updating. This suggests that persons with PSD were well able to inhibit salient but irrelevant reward information to update their beliefs.

### Relationship with cognition

Cognitive performance in several domains was lower in PSD as compared to HC and correlated with higher self-reported delusional ideation in the PDI. Yet, the relationship between task performance and delusional ideation remained significant when controlling for cognitive functioning in domains of verbal memory and information processing speed, while the CPP-difference between HC and PSD did not remain significant. It is not surprising that computations of CPP heavily rely on other cognitive functions. Inferring environmental dynamics is a cognitively demanding task and environmental volatility increases the level of difficulty to track outcomes. Furthermore, studies modelling belief perseveration^25,26^ showed that perseverance correlates particularly with cognitive performance. Therefore, the alterations were suggested to be non-specific to psychosis, but rather a cognitive performance deficit. Cognition was lower in several domains in PSD as compared to HC and correlated with higher self-reported delusional ideation. Others proposed that reinforcement learning deficits in schizophrenia may be at least partially explained by working memory deficits.^26^ Certainly, reinforcement learning tasks, such as the here adopted predictive inference task, cannot be decoupled from working memory. We tried to minimize the demand by initializing the cursor on every trial at the exact position of the participant belief from the previous trial. Our data supports that stronger neural encoding of CPP-dynamics is associated with higher verbal memory and information processing speed. This confirms prior work that neural circuits for cognitive domains and reinforcement learning are largely overlapping and work synergistically.^55^

### Limitations and future directions

The present study has several limitations: First, the groups were not matched in terms of age and the PSD group was relatively small. Second, although group differences were not significant we refrained from analysis controlling for gender. Third, the degree of self-reported delusional ideation clustered in the two groups, with relatively low scores in HC. This limits the validity of dimensional analysis with delusional ideation. Future studies should include people with high risk for psychosis to enhance variance on the scores. The PSD sample consisted of people with heterogeneous diagnoses on the psychosis spectrum. On one side, this may increase the external validity of our results. On the other hand, we cannot rule out that different diagnoses or disease stages result in differential behavioural or neural learning alterations that are blurred by aggregating them within a single group. We can also not rule out that our findings are unspecific to psychosis, as uncertainty-based belief updating seems to be altered in anxiety and depression too^56^ and as discussed above to general cognitive functioning, even in our study. Critically, our findings regarding group-differences and dimensional correlation with symptoms did not converge, just like relationships with ‘positive symptoms’ as measured with PDI versus PANSS. For instance, we found lower use of CPP in PSD versus HC on the group level, while results on the individual level suggested that CPP and ‘positive symptoms’ were only trend-wise associated, when measured with PDI across the whole sample, or not associated at all when measured with PANSS-positive in the PSD group. Divergence between PDI and PANSS scores could be partly explained by different content of the scales and different ‘time reference’ for symptoms (lifetime versus past week). Differences between results on the group versus individual level may be explained by afore-mentioned cognitive deficits, since significant group differences in CPP did not remain significant when controlling for cognitive function. We did not control whether perseveration resulted from a motivational deficit. The trackball device required effort and vigorous hand movement to move the cursor on the screen. This may have resulted in the observed neural activation of motor regions. Future studies could work with devices that require less motor effort. Another factor potentially contributing to alter belief updating is the antipsychotic medication that dampens dopamine transmission and may impact the observed behaviour. Yet, olanzapine equivalents did not correlate with performance, average LR and VOI-values from clusters that encoded CPP and RU and differed between groups (Supplementary material S11). Another limitation of the present study may be that the paradigm is relatively agnostic to typical content of hallucinations and delusions. As such, social components to target paranoid cognitions could be implemented in the paradigm as done by others.^57^ This would allow to investigate whether the learning alterations we found are even more pronounced when specific content is salient. Lastly, a limitation of our analysis approach is that we did not explore other models that may have explained the patient behaviour better. On the other hand, our current approach is similar to previous modelling studies^25,36,44,58^ and allows direct comparison across samples. Future studies could use model comparison to decide for a computational model. Moreover, to compare results from Gaussian Hierarchical Filter models it would be of interest to explore how certain parameters compare to the approximately Bayesian model adopted in the present study. Ideally, it could be explored in a single sample whether neural activation in response to comparable parameters from each model converges on similar regions.

## Conclusion

To our knowledge, this study is the first to examine encoding of RU and CPP in PSD. People with PSD showed lower performance and altered belief updating behaviour. They tended to react less responsive to environmental change points but instead persevered on previous beliefs and seemed to disregard the probability of environmental change points, in favour of the estimated relative belief uncertainty. Neural representations of these updating parameters were lower in people with PSD as compared to the control group. Interestingly, group differences were most pronounced in visual and motor areas, suggesting that people with PSD show altered integration of visual surprise signals and transmission to motor regions that guide behaviour.

## Supplementary Material

fcaf073_Supplementary_Data

## Data Availability

Data can be made available upon request if data protection regulations are fulfilled. Analysis scripts can be found at https://github.com/agschlagenhauf/DoReMe.git.
