# Supplementary material for: Neural correlates of uncertainty processing in psychosis spectrum disorder
Source: Brain Commun. 2025 Feb 17;7(1):fcaf073. doi: 10.1093/braincomms/fcaf073 (PMC11879018; doi:10.1093/braincomms/fcaf073)
Supplement: fcaf073_Supplementary_Data [file fcaf073_supplementary_data.docx]

**Supplementary material**

**1 Flowchart of participant eligibility and drop-out**

**Supplementary Figure 1.** **Flowchart of participant exclusion.** SCID = The Structured Clinical Interview for the Diagnostic and Statistical Manual of Mental Disorders, Fifth Edition, MRI = magnetic resonance imaging, MR-scanner = magnetic resonance scanner.

Sample size was limited by financial and logistic constraints.

**2 Task instructions**

**German version**

Willkommen zu diesem Computerspiel. Wir werden es Ihnen jetzt erklären. Benutzen Sie bitte die rechte Pfeiltaste, um vorwärts zu blättern und die linke Pfeiltaste, um zurück zu blättern.

Sie werden gleich einen Helikopter sehen. Der Helikopter wirft manchmal Geldsäcke ab. Ihre Aufgabe ist es diese zu fangen. Versuchen Sie so viel Geld wie möglich zu gewinnen. Ihren Geldgewinn bekommen Sie am Ende ausgezahlt!

Der Helikopter bei klarem Himmel:

--- Bild des Helicopters ---

Der Joystick steuert den weißen Cursor als Eimer. Damit fangen Sie auf, was der Helikopter abwirft. Versuchen Sie später den Eimer direkt unter dem Helikopter zu

Sie haben später 3 Sekunden, um sich für eine Position zu entscheiden. Sie werden dann den Joystick drücken, um die Entscheidung zu bestätigen. Dann wird der Eimer schwarz und lässt sich nicht mehr bewegen.

Dann sehen Sie den Abstand zwischen Eimer und tatsächlicher Position:

--- Bild von Feedback und Vorhersagefehler ---

Entweder wirft der Helikopter einen Sack mit Geld ab. Wenn Sie diesen fangen, bekommen Sie das Geld nach Spielende ausgezahlt.

Oder der Helikopter wirft einen Sandsack ab. Dafür bekommen Sie kein Geld. Versuchen Sie trotzdem ihn zu fangen. Normalerweise bleibt der Helikopter ungefähr an der gleichen Stelle. Manchmal ändert er aber auch völlig den Ort:

Bei wolkigem Himmel funktioniert alles genauso. Probieren Sie mal!

**English translation**

Welcome to this computer game. We will now explain it to you. Please use the right arrow key to scroll forward and the left arrow key to scroll back.

You will see a helicopter in a moment. The helicopter sometimes drops money bags. Your task is to catch them. Try to win as much money as possible. You will receive your cash prize at the end!

The helicopter in a clear sky:

--- Picture of the helicopter ---

The joystick controls the white cursor as a bucket. Use it to catch what the helicopter drops. Later, try to place the bucket directly under the helicopter.

You will later have 3 seconds to decide on a position. You will then press the joystick to confirm the decision. The bucket will then turn black and can no longer be moved.

You will then see the distance between the bucket and the actual position:

--- Picture of feedback and prediction error ---

Either the helicopter drops a bag of money. If you catch it, you will receive the money at the end of the game.

Or the helicopter drops a sandbag. You don't get any money for this. Try to catch it anyway. The helicopter usually stays in roughly the same place. Sometimes, however, it changes its location completely:

If the sky is cloudy, everything works exactly the same. Give it a try!

**3 Visual output of participant behavior**

**Examples from included subjects**


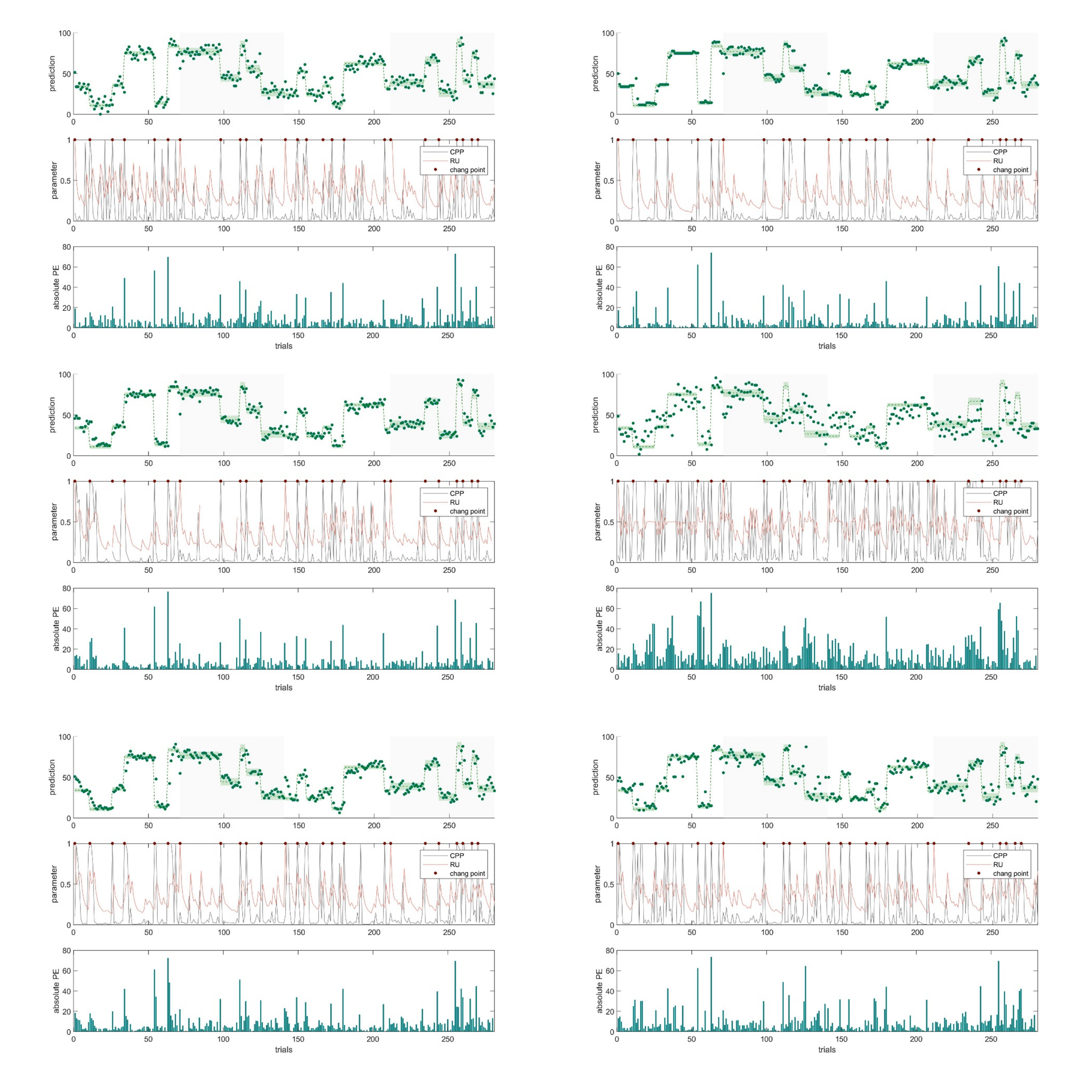


**Supplementary Figure 2.** **Examples of visual output from included patients with psychosis spectrum disorder.** Each mosaic shows the data of one participant. Across the three panels within each mosaic, the x-axis denotes the trials. On the top panel are participant predictions (dark green dots) and the represenation of the task distribution from which the bags were drawn, including the underlying mean (line) and standard deviation (shaded green). White and light gray background show the beginning and end of a block, after which participants took a break and when the standard deviation changes. On the middle panel are the respective parameter estimates of change point probabailtiy (CPP) and relative uncertainty (RU) from the computational model with true change points in the task (red dots). In the bottom are the absolute prediction errors (PEs) across the task.

These subjects behavior with predictions being close to the underlying mean, with the CPP and RU parameters increasing at true environmental change points and PEs being large in the beginning of a run and after change points.

**Excluded subjects**

**
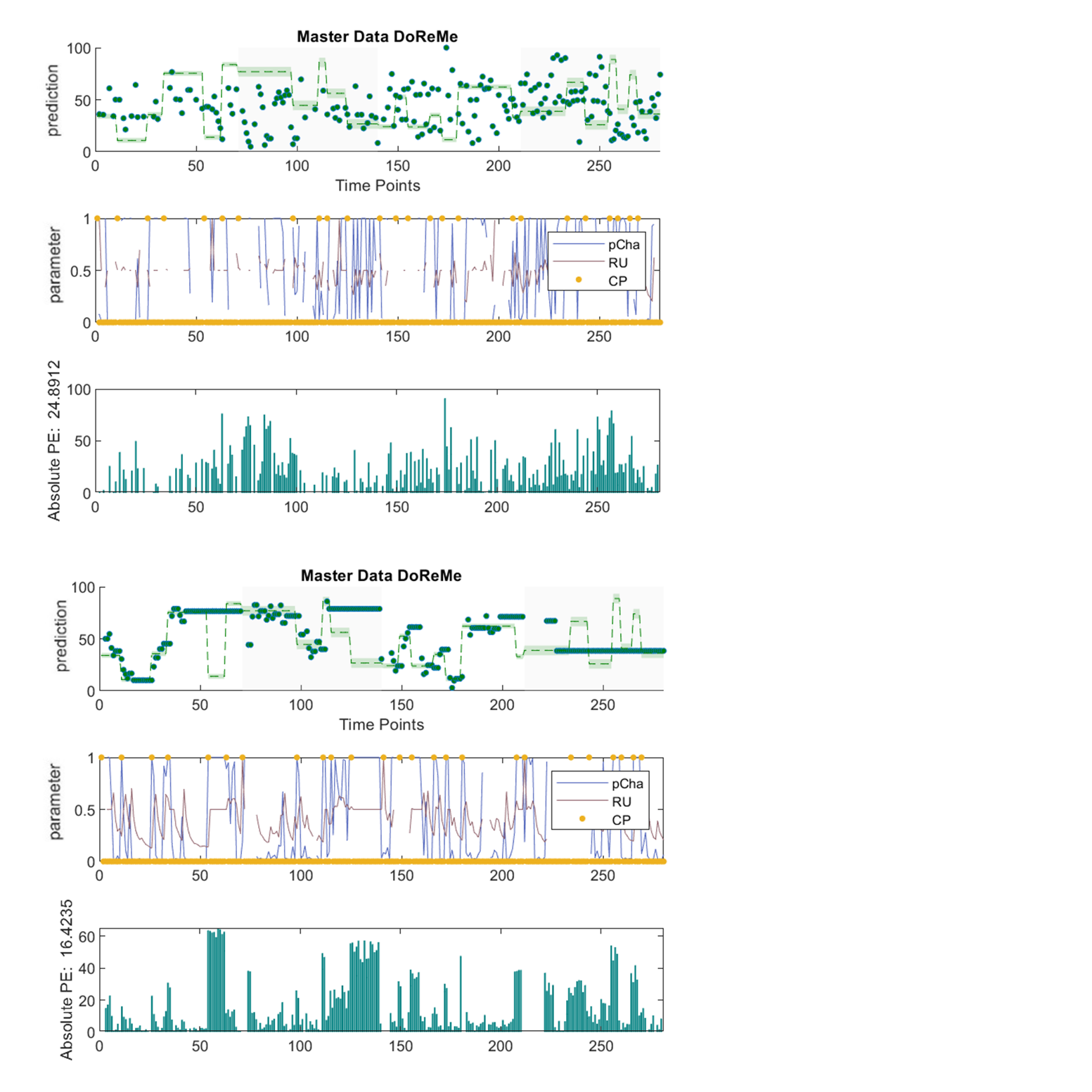
**

**Supplementary Figure 3. Examples of visual output from excluded patients with psychosis sptrum disorder.** Each mosaic shows the data of one participant. Across the three panels within each mosaic, the x-axis denotes the trials (time points). On the top panel are participant predictions (dark green dots) and the represenation of the task distribution from which the bags were drawn, including the underlying mean (line) and standard deviation (shaded green). White and light gray background show the beginning and end of a block, after which participants took a break and when the standard deviation changes. On the middle panel are the respective parameter estimates of change point probabailtiy (CPP) and relative uncertainty (RU) from the computational model with true change points in the task (yellow dots at the top). In the bottom are the absolute prediction errors (PEs) across the task.

This subject shows aberrant behavior with predictions being scattered across the whole screen and being unrelated to the outcomes. CPP and RU cannot be estimated properly and PEs are large across most of the task at true environmental change points and PEs being high across the task, regardless of change points.

**4 Computational model**

The computational model is similar to the model in McGuire et al. (2014). Belief updates${(B}_{t+1})$and prediction errors ${(\delta}_{t})$are computed from the observations $(X_{t})$ in form of a standard learning rule according to formula 1 and 2. Our computational model uses $\delta_{t}$ that were truly observed by the participants as inputs and computed a trialwise dynamic learning rate (𝛼𝑡), change point probability ($\Omega_{t}$) and relative uncertainty ($\tau_{t})$. The 𝛼𝑡 is dictated by $\Omega$ if a change point was likely (𝛺 ~ 1) and by $\tau_{t}$ if as change point is unlikely (𝛺 ~ 0).

$$\left( 1 \right) B_{t+1}=B_{t}+\alpha_{t}\times\delta_{t}$$

$${\left( 2 \right) \delta}_{t}=X_{t}-B_{t}$$

(3) 𝛼𝑡 = 𝛺 + 𝜏 – 𝛺𝜏

$\Omega_{t}$ is high when the likelihood of the most recent observations $X_{t}$ to occur under the current Gaussian belief distribution $N\left( X_{t}B_{t},\sigma_{t}^{2} \right)$ (with the mean at the inferred location and the standard deviation on the predictive distribution over future bag locations) is low as compared to a normal distribution across the whole outcome space (in our task 100 units). A normal distribution is assumed when a change point occurred because then the helicopter could be located everywhere on the screen with the same probability. The model is equipped with a fixed hazard rate that equaled the true rate at which change points took place in our task structure (H = .125). Change point probability increases in proportion to H, which models how frequent an agent that assumes change points to take place.

(4) $\Omega_{t}=\frac{U\left( X_{t}\mid0,100 \right)H}{U\left( X_{t}\mid0,100 \right)H+N\left( X_{t}B_{t},\sigma_{t}^{2} \right)\left( 1-H \right)}$

The relative uncertainty denotes the fraction of total uncertainty about the next observation resulting from an imprecise estimate of the helicopter location versus uncertainty resulting from noise: i.e. variance of the Gaussian distribution from which bag locations are sampled.

(5) $\tau_{t+1}=\frac{\Omega_{t}\sigma_{N}^{2}+\left( 1-\Omega_{t} \right)\tau_{t}\sigma_{N}^{2}+\Omega_{t}\left( 1-\Omega_{t} \right)\left( \delta_{t}\left( 1-\tau_{t} \right) \right)^{2}}{\Omega_{t}\sigma_{N}^{2}+\left( 1-\Omega_{t} \right)\tau_{t}\sigma_{N}^{2}+\Omega_{t}\left( 1-\Omega_{t} \right)\left( \delta_{t}\left( 1-\tau_{t} \right) \right)^{2}+\sigma_{N}^{2}}$

**5 Validation of group difference in performance errors**

To back up the result that PSD showed larger performance errors than HC, we conducted a rank-based Wilcoxon test. The Wilcoxon test confirmed previous results, suggesting significantly larger performance errors in PSD (W = 233, *p* = 0.017). Based on the interquartile range, we identified two subjects in PSD as outliers. Therefore, we also repeated the parametric t-test after excluding these two outliers in the PSD group. Again, the t-test supports higher performance errors in PSD (t = -2.27, *p* = 0.033) as reported in the manuscript.

**6 Analysis of PDI-scores on performance errors controlled for cognitive functioning**

Brief assessment of cognition in schizophrenia (BACS) symbol coding data of one healthy subject and PDI data of another healthy subject is missing, resulting in 57 observations.

**Supplementary Table 1. Regression of performance on PDI, mediated by BACS scores of cognitive functioning.**

| Predictors | Estimates | Confidence interval | p |
| --- | --- | --- | --- |
| (Intercept) | -1.18 | -23.16 – 20.80 | 0.915 |
| BACS verbal memory | 0.06 | -0.21 – 0.32 | 0.673 |
| BACS symbol coding | -0.13 | -0.33 – 0.06 | 0.176 |
| performance | 1.98 | 0.04 – 3.93 | 0.046 |
| Observations | 57 |  |  |
| R2 / R2 adjusted | 0.156 / 0.109 |  |  |

**7 Repetition of learning rate (LR) analysis after exclusion of trials with LR < -.1**

**Supplementary Table 2. Fraction of learning rate categories and group difference between HC and PSD.**

|  | HC | PSD | group difference |
| --- | --- | --- | --- |
| non-updates | 14.5% | 21% | t = 1.45, *p* = .156 |
| moderate updates | 42.7% | 38.5% | t = -1.37, *p* = .178. |
| total updates | 42.7% | 40.5% | t = -.45, *p* = .654 |


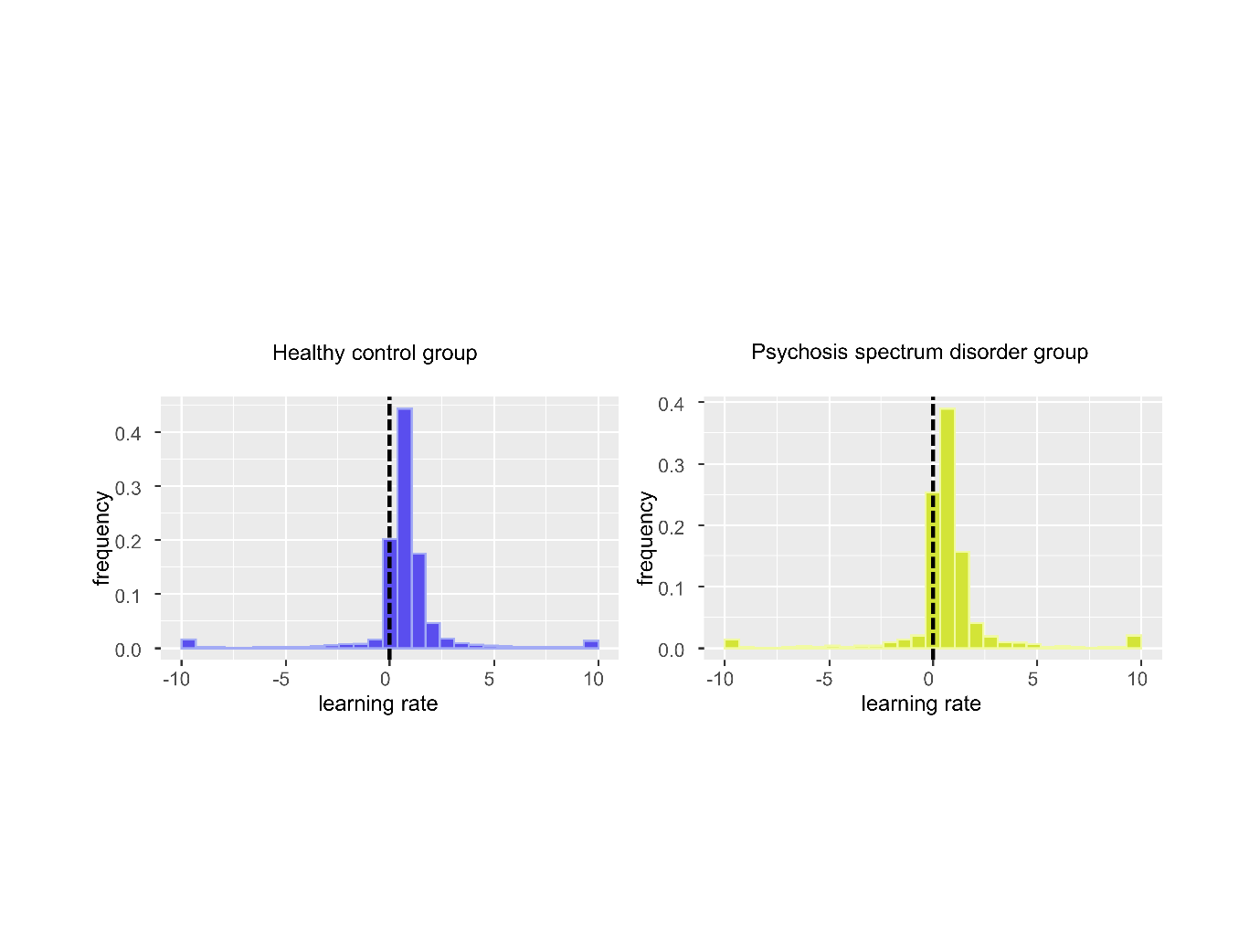


**Supplementary Figure 4. Histogram of learning rates (LR).** Panels show the healthy control group (n = 40) and patients with psychosis spectrum disorder (n = 19). For visualization, LR >10 are set to 10 and LR < -10 are set to -10.

**8 Distribution of regression parameters**

**
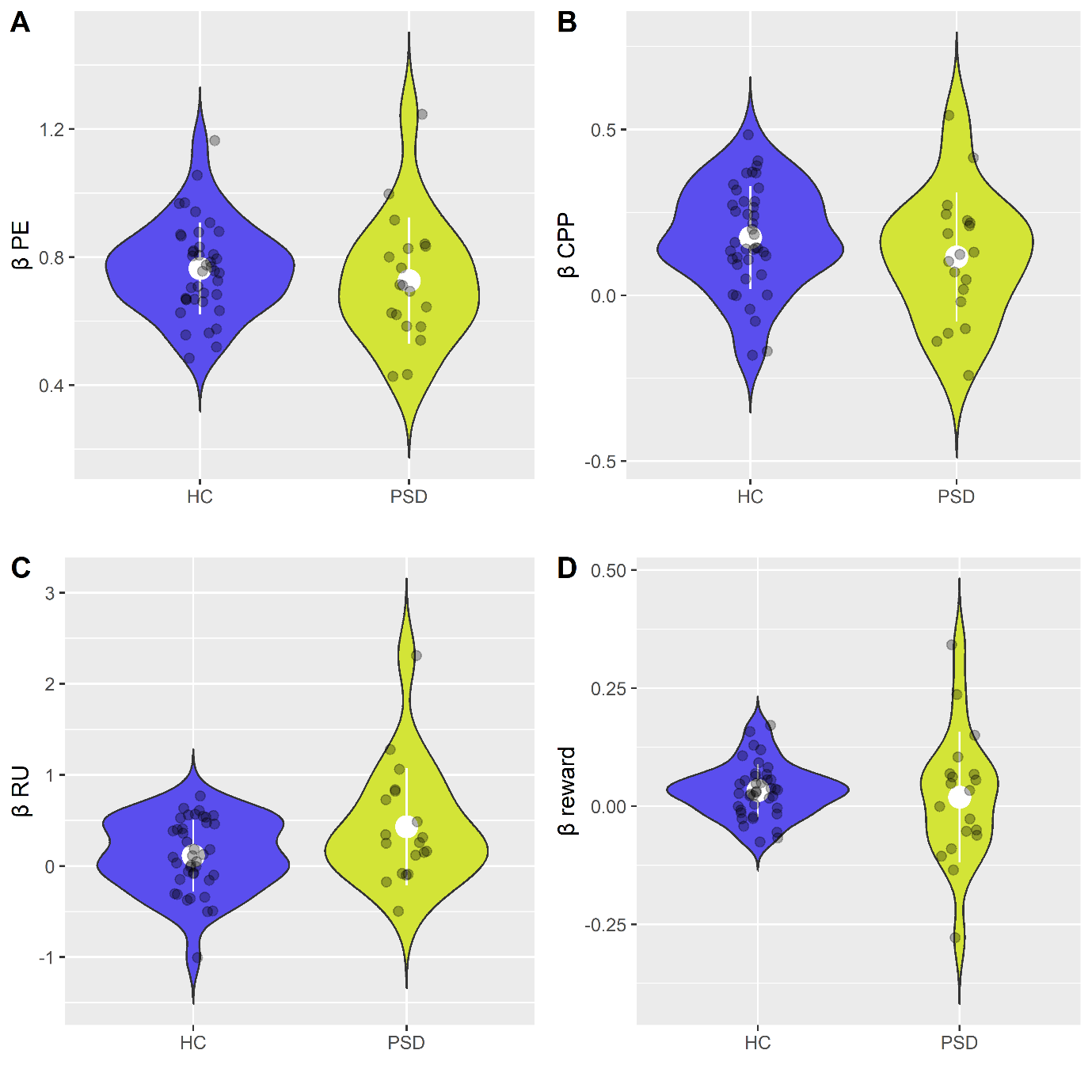
**

**Supplementary Figure 5. Distribution of regression model parameters.** Violines represent the distribution and dots represent individual participants. (HC = healthy control, n = 40; PSD = patients with psychosis spectrum disorder, n = 19). A) Prediction error (PE) of HC (*Md* = .76, *SD* = .14) and PSD (*Md* = .73, *SD* = .2). B) Change point probability (CPP) of HC (*Md* = .15, *SD* = .16) and PSD (*Md* = .12, *SD* = .19). C) Relative uncertainty (RU) of HC (*Md* = .11, *SD* = .39) and PSD (*Md* = .26, *SD* = .64). D) Reward of HC (*Md* = .03, *SD* = .06) and PSD (*Md* = .02, *SD* = .14). By means of a logistic regression approach we predicted group status from the regression model parameters. PE-magnitude was a trend-wise predictor (β = -5.4, z = -1.8, *p* = .072) and CPP a significant predictor of group status (β = -7.05, z = -2.33, *p* = .02), suggesting that updates of persons with PSD were less informed by PE-magnitude and CPP. Updating based on RU and reward had no predictive information for group status (β = -2.52, z = -.6, *p* = .551).

**9 Analysis of group status on model parameters controlled for cognition functioning**

BACS symbol coding data of one healthy subject is missing, resulting in 58 observations.

**Supplementary Table 3. Logistic regression of model coefficients on group (HC/PSD), mediated by BACS scores of cognitive functioning. (PE = Prediction error, CPP = Change point probability, RU = Relative uncertainty).**

| Predictors | Odds Ratios | Confidence interva | *p* |
| --- | --- | --- | --- |
| β PE | 0.00 | 0.00 – 3.47 | 0.127 |
| β PE CPP | 0.00 | 0.00 – 4.06 | 0.133 |
| β PE RU | 5.24 | 0.95 – 47.74 | 0.091 |
| β PE reward | 13.90 | 0.00 – 1006026.52 | 0.629 |
| BACS verbal memory | 0.90 | 0.80 – 1.00 | 0.062 |
| BACS symbol coding | 0.97 | 0.90 – 1.03 | 0.311 |
| Observations | 58 |  |  |
| R2 Tjur | 0.345 | | |
| (Intercept) | 57524.32 | | |

**10 Neural activation related to model parameters and reward**

**Supplementary Table 4. Positive activation related to CPP (FWE-whole brain corrected, cluster-threshold k > 10).**

| **cluster-level** | | | | **peak-level** | | | | |  |  |  |
| --- | --- | --- | --- | --- | --- | --- | --- | --- | --- | --- | --- |
| pFWEcorr | pFDRcorr | equivk | punc | pFWEcorr1 | pFDRcorr1 | T | equivZ | punc1 | x | y | z |
|  |  |  |  |  |  |  |  |  |  |  |  |
|  | <. 001 | 28007 | 0 | <. 001 | <. 001 | 16,02 | 65535 | 0 | -41 | -21 | 52 |
|  |  |  |  | <. 001 | <. 001 | 15,4 | 65535 | 0 | -37 | -29 | 56 |
|  |  |  |  | <. 001 | <. 001 | 14,69 | 65535 | 0 | 22 | -59 | 24 |
| <. 001 | <. 001 | 467 | 0 | <. 001 | <. 001 | 10,45 | 65535 | 0 | 16 | -61 | -51 |
|  |  |  |  | <. 001 | 0,004 | 6,28 | 6,11 | 0 | 36 | -49 | -49 |
| <. 001 | <. 001 | 228 | 0 | <. 001 | <. 001 | 10,12 | 65535 | 0 | -51 | -23 | 22 |
| <. 001 | <. 001 | 1149 | 0 | <. 001 | <. 001 | 9,86 | 65535 | 0 | -33 | 38 | 36 |
|  |  |  |  | <. 001 | <. 001 | 9,32 | 65535 | 0 | -29 | 40 | 28 |
|  |  |  |  | <. 001 | <. 001 | 7,09 | 6,84 | 0 | -45 | 32 | 36 |
| <. 001 | <. 001 | 507 | 0 | <. 001 | <. 001 | 9,56 | 65535 | 0 | -41 | 16 | 6 |
|  |  |  |  | <. 001 | <. 001 | 8,02 | 7,67 | 0 | -31 | 28 | 6 |
|  |  |  |  | <. 001 | <. 001 | 6,7 | 6,48 | 0 | -31 | 20 | -9 |
| <. 001 | <. 001 | 984 | 0 | <. 001 | <. 001 | 9,29 | 65535 | 0 | 32 | 42 | 30 |
|  |  |  |  | <. 001 | <. 001 | 7,05 | 6,81 | 0 | 32 | 38 | 46 |
|  |  |  |  | <. 001 | <. 001 | 7,05 | 6,8 | 0 | 28 | 30 | 42 |
| <. 001 | <. 001 | 449 | 0 | <. 001 | <. 001 | 8,82 | 65535 | 0 | -17 | -21 | 8 |
|  |  |  |  | <. 001 | <. 001 | 7,09 | 6,85 | 0 | 6 | -23 | -1 |
|  |  |  |  | <. 001 | <. 001 | 6,97 | 6,73 | 0 | -5 | -23 | -5 |
| <. 001 | <. 001 | 270 | 0 | <. 001 | <. 001 | 8,57 | 65535 | 0 | -33 | -57 | -31 |
|  |  |  |  | <. 001 | <. 001 | 8,04 | 7,69 | 0 | -41 | -59 | -31 |
| <. 001 | <. 001 | 412 | 0 | <. 001 | <. 001 | 7,91 | 7,58 | 0 | 34 | 26 | 6 |
|  |  |  |  | <. 001 | <. 001 | 7,34 | 7,07 | 0 | 44 | 24 | 4 |
|  |  |  |  | <. 001 | <. 001 | 6,84 | 6,61 | 0 | 46 | 14 | 2 |
| <. 001 | <. 001 | 86 | 0 | <. 001 | <. 001 | 7,45 | 7,17 | 0 | -47 | 2 | 8 |
| <. 001 | <. 001 | 79 | 0 | <. 001 | <. 001 | 7,38 | 7,11 | 0 | -13 | -53 | -51 |
|  |  |  |  | 0,009 | 0,194 | 5,45 | 5,34 | 0 | -11 | -55 | -59 |
| <. 001 | <. 001 | 104 | 0 | <. 001 | 0,001 | 6,63 | 6,42 | 0 | -21 | 12 | 6 |
|  |  |  |  | 0,002 | 0,048 | 5,76 | 5,62 | 0 | -23 | 2 | 4 |
| <. 001 | <. 001 | 65 | 0 | <. 001 | 0,002 | 6,4 | 6,22 | 0 | 48 | -21 | 44 |
| <. 001 | <. 001 | 33 | 0 | <. 001 | 0,012 | 6,05 | 5,89 | 0 | 54 | -25 | -9 |
| <. 001 | <. 001 | 52 | 0 | <. 001 | 0,013 | 6,03 | 5,87 | 0 | -41 | -53 | -47 |
| <. 001 | 0,003 | 17 | 0,002 | 0,001 | 0,02 | 5,94 | 5,79 | 0 | 26 | 60 | 10 |
| <. 001 | <. 001 | 34 | 0 | 0,001 | 0,021 | 5,93 | 5,78 | 0 | -57 | -43 | 36 |
|  |  |  |  | 0,02 | 0,411 | 5,28 | 5,17 | 0 | -65 | -41 | 32 |
| <. 001 | <. 001 | 55 | 0 | 0,001 | 0,022 | 5,92 | 5,77 | 0 | -61 | -55 | 18 |
|  |  |  |  | 0,003 | 0,07 | 5,68 | 5,55 | 0 | -49 | -51 | 14 |
| <. 001 | 0,001 | 23 | 0,001 | 0,001 | 0,022 | 5,92 | 5,77 | 0 | 6 | -69 | -37 |
| <. 001 | 0,001 | 24 | 0 | 0,001 | 0,034 | 5,83 | 5,69 | 0 | 32 | 24 | -11 |

**Supplementary Table 5. Negative activation related to CPP (FWE-whole brain corrected, cluster-threshold k > 10).**

| **cluster-level** | | | | **peak-level** | | | | |  |  |  |
| --- | --- | --- | --- | --- | --- | --- | --- | --- | --- | --- | --- |
| pFWEcorr | pFDRcorr | equivk | punc | pFWEcorr1 | pFDRcorr1 | T | equivZ | punc1 | x | y | z |
|  |  |  |  |  |  |  |  |  |  |  |  |
| 0,001 | 0,027 | 11 | 0,011 | <. 001 | 0,027 | 6,26 | 6,09 | 0 | 10 | 12 | -7 |
| <. 001 | 0,003 | 22 | 0,001 | <. 001 | 0,027 | 6,23 | 6,06 | 0 | -9 | 12 | -5 |
| <. 001 | 0,001 | 32 | 0 | <. 001 | 0,027 | 6,18 | 6,01 | 0 | 36 | -87 | -7 |
| <. 001 | 0,001 | 34 | 0 | 0,001 | 0,042 | 6,01 | 5,86 | 0 | -17 | -17 | 30 |
| <. 001 | 0,012 | 15 | 0,004 | 0,001 | 0,08 | 5,85 | 5,71 | 0 | -39 | 34 | -15 |
| <. 001 | 0,003 | 23 | 0,001 | 0,003 | 0,157 | 5,68 | 5,55 | 0 | -23 | -47 | 18 |
|  |  |  |  | 0,016 | 0,552 | 5,32 | 5,21 | 0 | -29 | -55 | 14 |

**
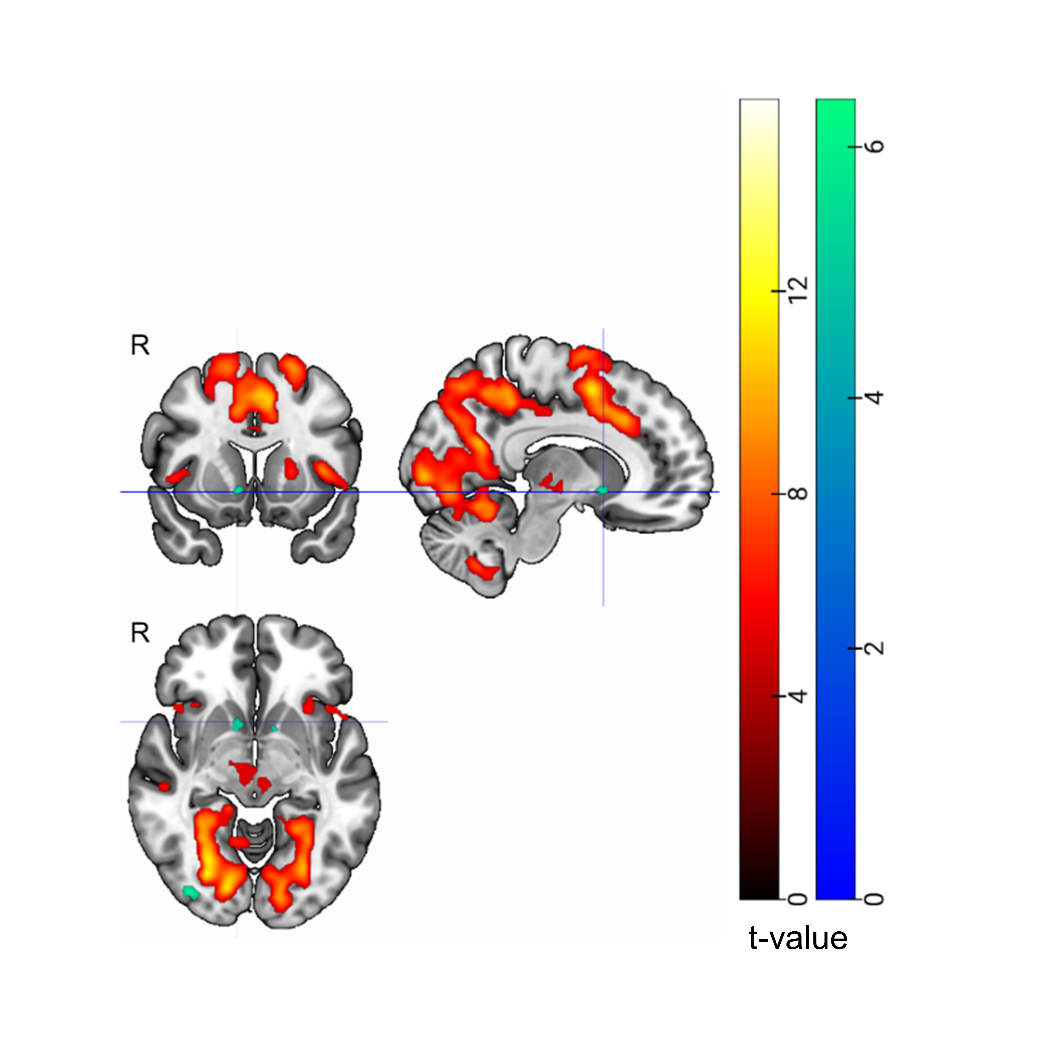
**

**Supplementary Figure 6. Neural activation to change point probability (CPP).** Red shows positive and blue shows negative activation across all participants n = 59, (displayed at xyz[10, 12, -7], cluster-level FWE-corrected p < 0.05, k > 10). Color bar and heatmaps represent t-statistics of an one-sample t-test.

**Supplementary Table 6. Positive activation related to RU (FWE-whole brain corrected, cluster-threshold k > 10).**

| **cluster-level** | | | | **peak-level** | | | | |  |  |  |
| --- | --- | --- | --- | --- | --- | --- | --- | --- | --- | --- | --- |
| pFWEcorr | pFDRcorr | equivk | punc | pFWEcorr1 | pFDRcorr1 | T | equivZ | punc1 | x | y | z |
| <. 001 | <. 001 | 8478 | 0 | <. 001 | <. 001 | 15,38 | 65535 | 0 | 20 | -53 | -25 |
|  |  |  |  | <. 001 | <. 001 | 14,14 | 65535 | 0 | 4 | -63 | -19 |
|  |  |  |  | <. 001 | <. 001 | 13,81 | 65535 | 0 | 18 | -61 | -47 |
| <. 001 | <. 001 | 4886 | 0 | <. 001 | <. 001 | 11,24 | 65535 | 0 | -37 | -23 | 70 |
|  |  |  |  | <. 001 | <. 001 | 9,92 | 65535 | 0 | -43 | -39 | 66 |
|  |  |  |  | <. 001 | <. 001 | 9,47 | 65535 | 0 | -47 | -35 | 54 |
| <. 001 | <. 001 | 499 | 0 | <. 001 | <. 001 | 7,26 | 6,99 | 0 | 50 | -29 | 46 |
|  |  |  |  | <. 001 | <. 001 | 6,88 | 6,65 | 0 | 48 | -35 | 60 |
|  |  |  |  | 0,003 | 0,09 | 5,66 | 5,53 | 0 | 56 | -37 | 56 |
| <. 001 | 0,001 | 26 | 0 | <. 001 | 0,001 | 6,64 | 6,44 | 0 | -3 | -37 | 78 |
| <. 001 | <. 001 | 49 | 0 | <. 001 | 0,002 | 6,53 | 6,33 | 0 | -5 | -7 | 78 |
|  |  |  |  | 0,002 | 0,054 | 5,78 | 5,64 | 0 | -5 | -23 | 80 |
| <. 001 | <. 001 | 295 | 0 | <. 001 | 0,002 | 6,52 | 6,33 | 0 | 36 | -7 | 66 |
|  |  |  |  | <. 001 | 0,012 | 6,11 | 5,94 | 0 | 22 | -3 | 50 |
|  |  |  |  | 0,001 | 0,033 | 5,89 | 5,74 | 0 | 30 | -11 | 52 |
| <. 001 | <. 001 | 162 | 0 | <. 001 | 0,004 | 6,33 | 6,15 | 0 | 32 | 60 | 20 |
|  |  |  |  | <. 001 | 0,013 | 6,09 | 5,93 | 0 | 36 | 46 | 26 |
|  |  |  |  | 0,003 | 0,073 | 5,71 | 5,58 | 0 | 40 | 52 | 20 |
| <. 001 | 0,001 | 27 | 0 | <. 001 | 0,005 | 6,3 | 6,12 | 0 | -29 | -21 | 10 |
| <. 001 | <. 001 | 108 | 0 | <. 001 | 0,006 | 6,27 | 6,09 | 0 | 54 | -41 | 34 |
|  |  |  |  | 0,025 | 0,55 | 5,23 | 5,13 | 0 | 50 | -45 | 42 |
| <. 001 | 0,002 | 23 | 0,001 | 0,001 | 0,033 | 5,89 | 5,74 | 0 | -11 | -81 | 54 |
| <. 001 | 0,02 | 12 | 0,008 | 0,002 | 0,048 | 5,8 | 5,66 | 0 | -19 | -23 | 20 |
| <. 001 | <. 001 | 34 | 0 | 0,003 | 0,09 | 5,66 | 5,53 | 0 | -17 | -23 | 6 |
| <. 001 | 0,003 | 20 | 0,001 | 0,006 | 0,151 | 5,54 | 5,41 | 0 | 36 | -85 | 28 |

**
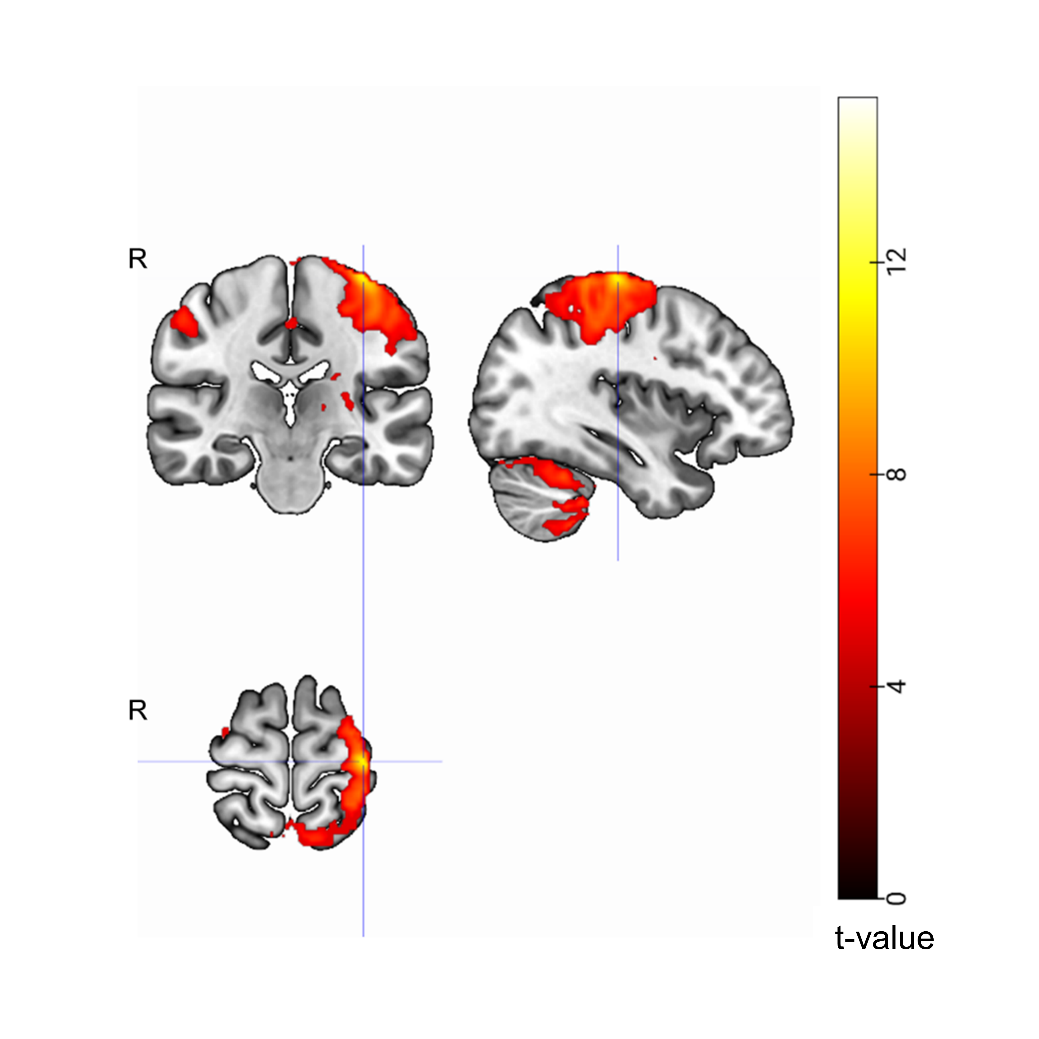
**

**Supplementary Figure 7. Neural activation relative uncertainty (RU).** Positive activation across all participants, n = 59 (displayed at xyz[-37, 23, 70], cluster-level FWE-corrected p < 0.05, k > 10). Color bar and heatmaps represent t-statistics of an one-sample t-test.

**Supplementary Table 7. Positive activation related to reward (p = .001, uncorrected, cluster-threshold k > 10).**

| **cluster-level** | | | | **peak-level** | | | | | | | |
| --- | --- | --- | --- | --- | --- | --- | --- | --- | --- | --- | --- |
| pFWEcorr | pFDRcorr | equivk | punc | pFWEcorr1 | pFDRcorr1 | T | equivZ | punc1 | x | y | z |
|  |  |  |  |  |  |  |  |  |  |  |  |
| <. 001 | <. 001 | 757 | 0 | <. 001 | <. 001 | 10,06 | 65535 | 0 | -37 | -87 | -13 |
|  |  |  |  | <. 001 | <. 001 | 8,31 | 65535 | 0 | -39 | -67 | -15 |
|  |  |  |  | <. 001 | <. 001 | 7,09 | 6,84 | 0 | -37 | -59 | -19 |
| <. 001 | <. 001 | 16 | 0 | <. 001 | <. 001 | 8,55 | 65535 | 0 | 32 | -91 | -11 |
|  |  |  |  | <. 001 | <. 001 | 7,2 | 6,94 | 0 | 38 | -81 | -11 |
|  |  |  |  | <. 001 | 0,001 | 6,81 | 6,59 | 0 | 36 | -73 | -13 |
| <. 001 | <. 001 | 132 | 0 | <. 001 | <. 001 | 7,56 | 7,27 | 0 | 10 | 14 | 8 |
|  |  |  |  | 0,015 | 0,377 | 5,34 | 5,23 | 0 | 12 | 6 | 16 |
| <. 001 | <. 001 | 213 | 0 | <. 001 | <. 001 | 7,46 | 7,17 | 0 | 30 | 22 | -15 |
|  |  |  |  | <. 001 | 0,002 | 6,53 | 6,33 | 0 | 34 | 22 | -7 |
| <. 001 | <. 001 | 68 | 0 | <. 001 | 0,001 | 6,79 | 6,57 | 0 | -11 | 10 | -1 |
| <. 001 | <. 001 | 64 | 0 | <. 001 | 0,002 | 6,58 | 6,38 | 0 | -29 | 20 | -11 |
|  |  |  |  | 0,002 | 0,062 | 5,79 | 5,65 | 0 | -33 | 24 | -3 |
|  |  |  |  | 0,009 | 0,254 | 5,46 | 5,34 | 0 | -43 | 20 | -7 |
| <. 001 | <. 001 | 44 | 0 | <. 001 | 0,002 | 6,52 | 6,32 | 0 | 6 | 34 | 48 |
| <. 001 | <. 001 | 104 | 0 | <. 001 | 0,006 | 6,32 | 6,14 | 0 | 8 | 40 | 22 |
|  |  |  |  | 0,008 | 0,227 | 5,49 | 5,37 | 0 | 4 | 40 | 36 |
| <. 001 | <. 001 | 57 | 0 | <. 001 | 0,02 | 6,04 | 5,88 | 0 | 10 | 42 | 8 |
| <. 001 | 0,015 | 12 | 0,008 | 0,002 | 0,069 | 5,75 | 5,61 | 0 | 52 | -37 | 48 |

**
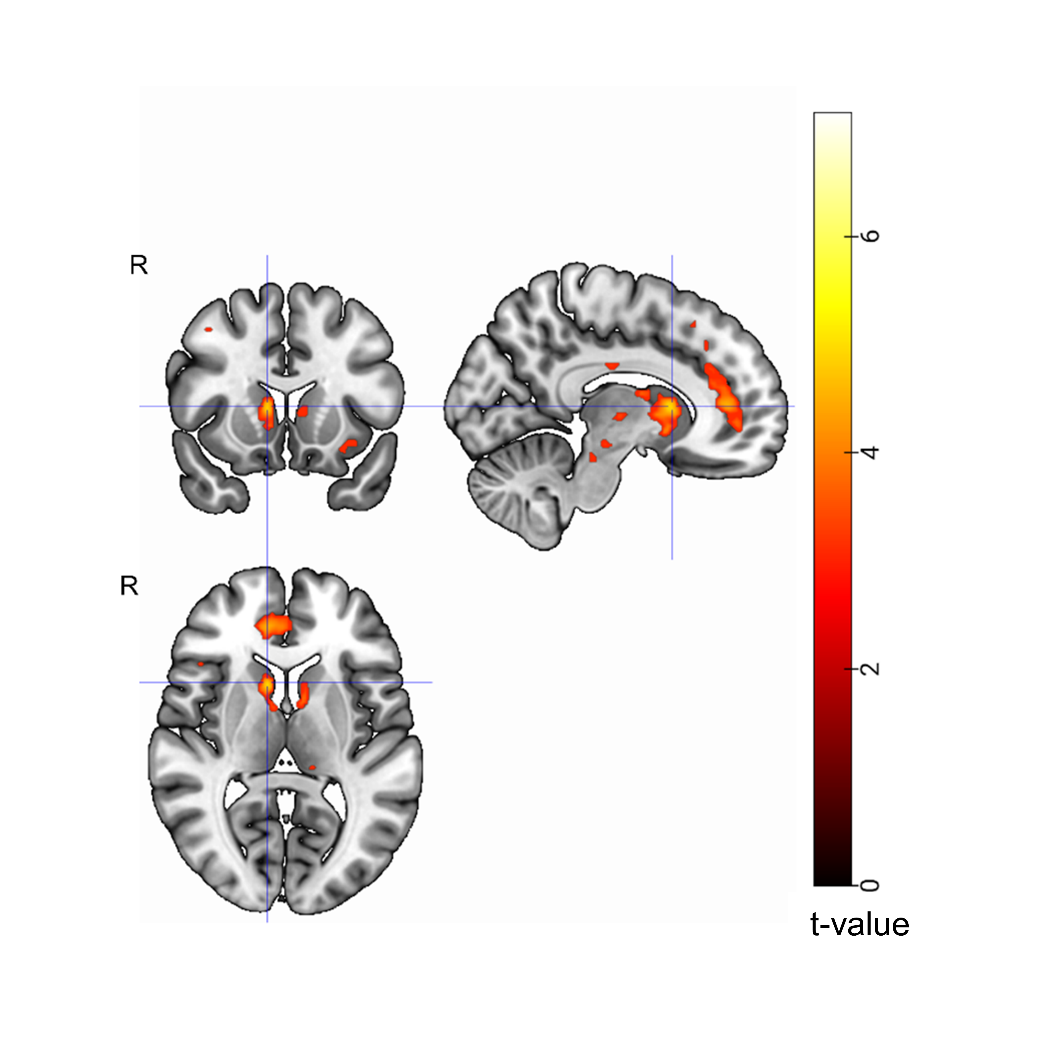
**

**Supplementary Figure 8. Neural activation related to reward*.*** Positive activation across all participants, n = 59 (displayed at xyz[10, 14, 8], *p* < 0.001 uncorrected, k > 10). Color bar and heatmaps represent t-statistics of an one-sample t-test.

**11 Relationship with medication**

Performance (rTAU =0.27, *p* = .116) and average learning rate across trials (rTAU= -0.13, *p* = .199) did not correlate with olanzapine equivalents. VOI values from the clusters that encoded change point probability and relative belief uncertainty and differed between groups did not correlate with the olanzapine equivalents (all *p* > .01).
